# Whole-Genome Sequencing of Measles Virus from 2025 Outbreak, Texas, USA 

**DOI:** 10.3201/eid3210.260494

**Published:** 2026-10

**Authors:** Piyada Juntawong, Bonnie Oh, Richard Bovio, Jie Lu, Sydney Stanley, Robert F. Potter, Yan Sun, Casey Schroeder, Olivia A. Smith, Elise Huebner, Stephen L. White, Saroj Rai, Varun Shetty, Grace Kubin

**Affiliations:** Texas Department of State Health Services, Austin, Texas, USA (P. Juntawong, B. Oh, R. Bovio, J. Lu, Y. Sun, C. Schroeder, O.A. Smith, E. Huebner, S.L. White, S. Rai, V. Shetty, G. Kubin); Children's Hospital of Philadelphia, Philadelphia, Pennsylvania, USA (S. Stanley, R.F. Potter); Perelman School of Medicine at the University of Pennsylvania, Philadelphia (S. Stanley, R.F. Potter)

**Keywords:** measles virus, whole-genome sequencing, viruses, genomic surveillance, outbreak investigation, genotype D8, phylogenetics, United States

## Abstract

We sequenced measles-positive samples from the 2025 outbreak in Texas, USA, and identified the expansion of a closely related genotype D8 lineage within broader strain diversity from North America. F:H419R was used as a lineage-associated genomic surveillance marker. Our findings demonstrate that whole-genome sequencing can strengthen measles surveillance and enhance outbreak investigations.

Measles virus importations continue to cause outbreaks in undervaccinated communities in the United States despite elimination since 2000 (*1*). In early 2025, the Texas Department of State Health Services reported the state’s largest measles outbreak after elimination, centered in Gaines County and neighboring West Texas communities (*2*). Epidemiologic reports indicated 762 confirmed measles cases; more than half occurred in Gaines County, and most cases were among children, adolescents, and persons who were unvaccinated or had unknown vaccination status (*3*). The outbreak occurred during expanded measles activity in North America, raising questions about local expansion, interstate relatedness, and the utility of whole-genome sequencing (WGS) to resolve transmission patterns beyond routine genotyping.

Routine measles molecular surveillance commonly relies on the 450-nucleotide region of the nucleoprotein gene (N-450), which supports genotype and distinct sequence identifier (DSId) assignment. However, N-450 provides limited resolution of transmission chains during large outbreaks involving closely related viruses (*4*). We used WGS to characterize the measles outbreak in Texas and assessed genomic diversity and genomic relatedness to other measles virus genomes from North America. 

## The Study

We performed tiled amplicon-based sequencing on 491 reverse transcription PCR–positive measles samples (Appendix). After quality assessment, 368 measles virus genomes from 359 persons met the predefined coverage threshold for phylogenetic analysis; 9 persons each had 2 samples sequenced (Appendix Table 1). Among the 359 persons with sequenced samples and epidemiologic metadata, 204 (56.8%) were unvaccinated, 139 (38.7%) had unknown vaccination status, 7 (2.0%) had received 1 measles vaccine dose, and 9 (2.5%) had received 2 measles vaccine doses (Appendix Table 2). We determined vaccination status by using patient records. Patients without confirmed vaccination status were classified as unknown. We performed an exploratory genome-wide association study to evaluate if measles variants were associated with vaccination status, but we did not detect any variants significantly associated with vaccination status; however, our analysis was underpowered. 

We performed a maximum-likelihood phylogenic analysis by using the 368 sequenced measles virus genomes. N-450 DSIds were available for 240 measles virus genomes, of which 220 (92%) were DSId 9171, a specific measles virus lineage belonging to the D8 genotype. We assigned epidemiologic weeks to measles virus genomes by using Morbidity and Mortality Weekly Report numbering (*5*). Maximum-likelihood phylogenetic analysis revealed that measles virus genomes from Texas formed a closely related genotype D8 lineage (Figure 1, panel A). Pairwise single-nucleotide polymorphism (SNP) distances ranged from 0–15 SNPs (median 3 SNPs), supporting close genetic relatedness among outbreak genomes (Figure 1, panel B). 

**Figure 1:**
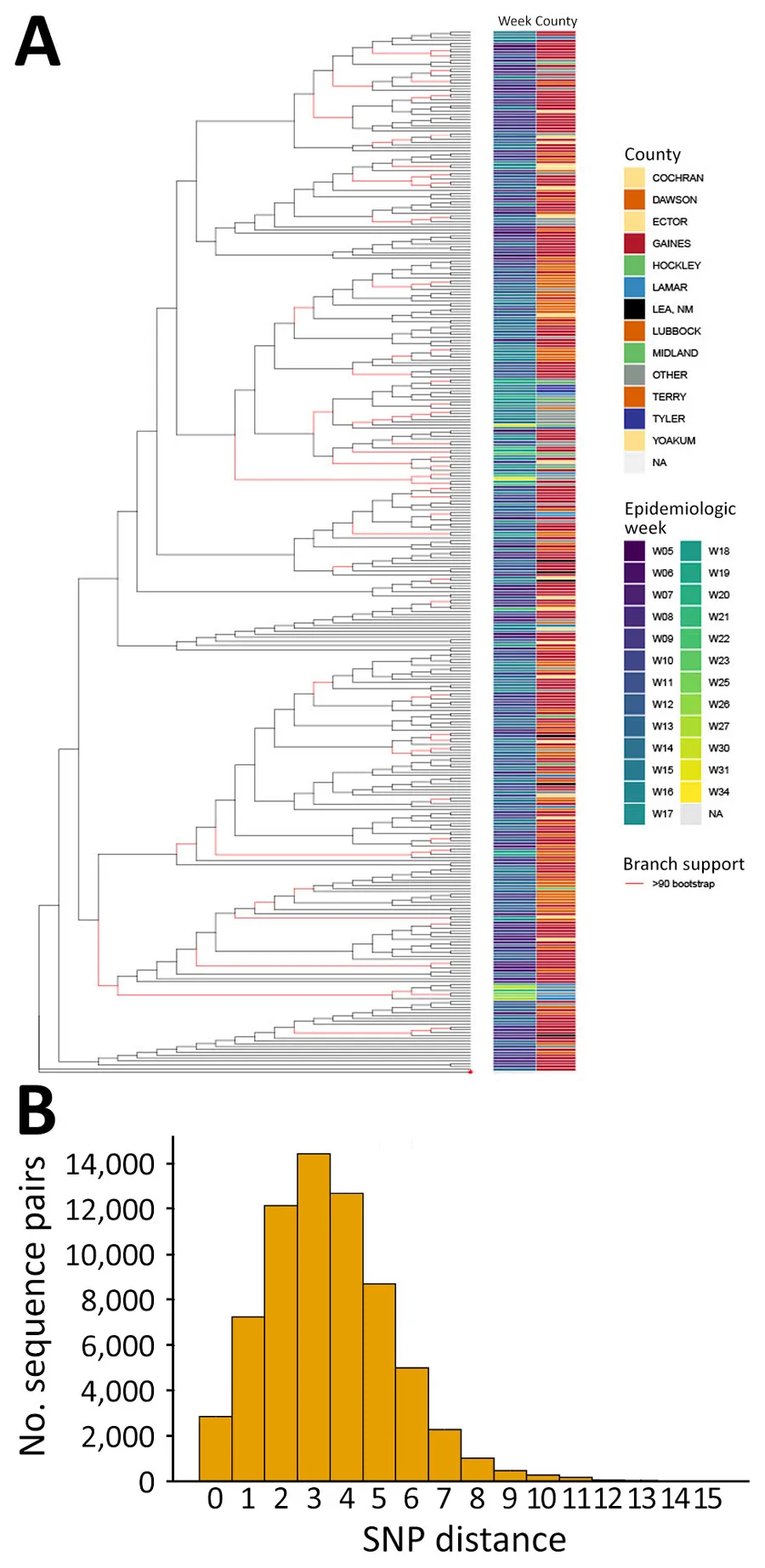
Genomic diversity of measles virus genomes from study of whole-genome sequencing of measles virus from 2025 outbreak, Texas, USA. A) Maximum-likelihood phylogenetic tree of 368 Texas measles genomes (January–August 2025). Color strip annotations denote epidemiologic week of specimen collection and county. Red branches indicate clusters with an ultrafast bootstrap value >90. Red asterisk indicates reference D8 strain (GenBank accession no. KJ018971.2). B) Distribution of pairwise SNP distances among Texas measles genomes. SNP, single-nucleotide polymorphism.

To estimate the time to most recent common ancestor (tMRCA) for the measles virus genomes associated with the outbreak in Texas, we performed a molecular clock analysis. We did not use tMRCA estimates from the Texas measles virus genomes alone to infer introduction timing because our molecular clock analysis revealed weak temporal signal after we excluded the temporally distant D8 reference genome (Appendix Figure 1). Early measles virus genomes from epidemiologic weeks 5–8 were concentrated in Gaines County, Texas, the outbreak epicenter. We later observed closely related genomes in nearby and more distant Texas counties (Appendix Figure 2). Those results support early localized amplification followed by wider spread, although multiple closely related introductions cannot be ruled out (*6*).

To provide genomic surveillance context, we conducted a targeted evaluation of the Fusion gene H419R substitution (F:H419R), fixed in all measles viral genomes from Texas, rather than a comprehensive genome-wide screen for recurrent, homoplasic substitution, or substitutions of functional effect. To place F:H419R in broader context, we screened publicly available genotype D8 whole genomes with adequate Fusion gene coverage (>12,500 bp of total genome coverage with an unambiguous call for the Fusion gene codon encoding residue 419). We detected F:H419R among recent publicly available D8 measles virus genomes (Figure 2, panel A; Appendix Table 3). The proportion of D8 genomes with the F:H419R substitution increased from 2% (1/49) in 2023 to 8.5% (10/118) in 2024 and 65.4% (34/52) in 2025 (Appendix Figure 3). Although residue 419 has prior residue-level annotation (*7*), this study did not evaluate functional, immunologic, vaccine-effectiveness, transmission, or clinical consequences. Selection analysis identified 2 polymerase-gene codons, which we interpreted as subclade markers rather than evidence of immune escape or functional adaptation (Appendix Figure 4).

**Figure 2:**
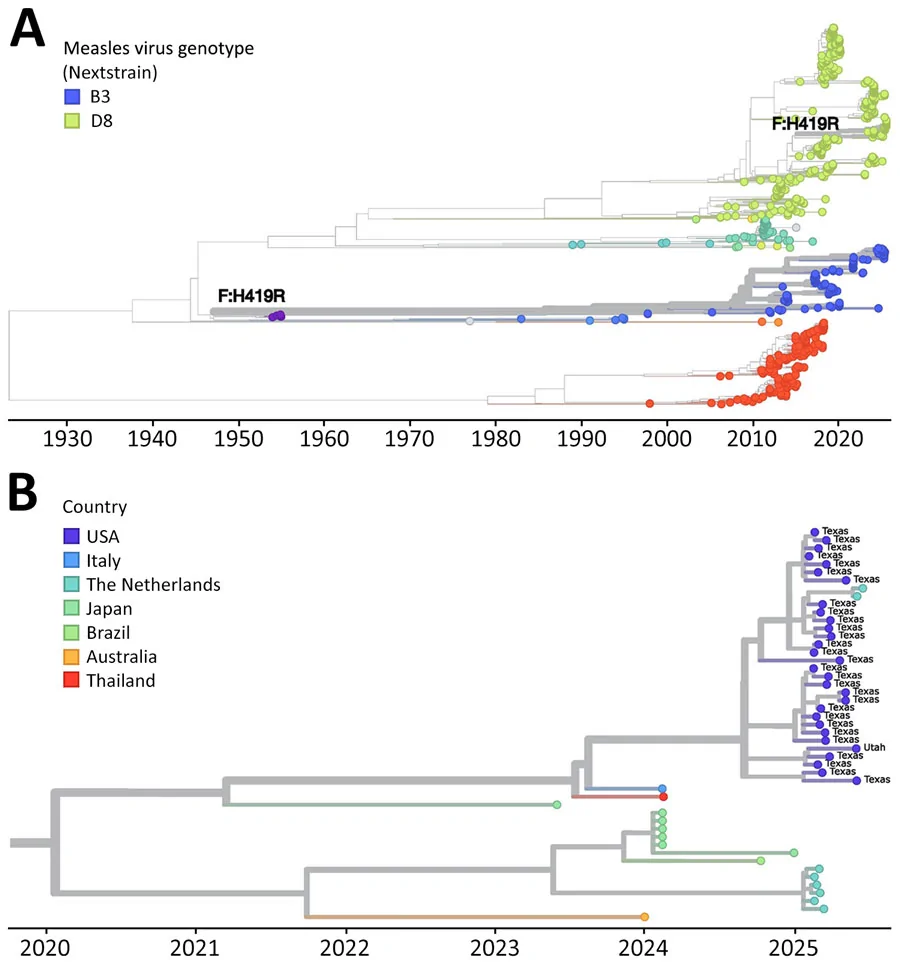
Genomic surveillance context of F:H419R among publicly available measles virus genomes from study of whole-genome sequencing of measles virus from 2025 outbreak, Texas, USA. A) Nextstrain time-scaled global phylogeny of measles viruses showing genotype B3 and D8 measles virus genomes with the F:H419R substitution highlighted (https://nextstrain.org/measles/genome@2025-09-24?gt=F.419R&treeZoom=selected). B) Global genotype D8 context of representative measles genomes associated with the outbreak in Texas and representative publicly available F:H419R-positive D8 genomes. F:H419R, Fusion gene H419R substitution.

We placed the measles genomes from Texas in the global context by using the Nextstrain measles workflow (https://github.com/nextstrain/measles). To reduce geographic and temporal sampling imbalance, we subsampled genomes to <30 per country–year group. Consequently, the phylogeny includes only a representative subset of 2025 measles genomes from the United States (Figure 2, panel B). Measles virus genomes from Texas clustered with contemporaneous genomes from Utah, USA, and the Netherlands (Figure 2, panel B). 

We analyzed a comparative dataset including genomes from Texas, Arizona, Utah, South Carolina, and other jurisdictions in North America. We observed high callable coverage across most coding regions including N-450 and the Fusion gene but more variable coverage in the matrix–fusion noncoding region (MF-NCR) (Appendix Figure 5). Masking MF-NCR positions did not alter major phylogenetic clustering, indicating that uneven coverage did not account for the broad phylogenetic structure (Appendix Figure 6). The focal measles virus genomes from North America were interspersed with other related D8 measles virus genotypes (Figure 3). Root-to-tip regression indicated limited temporal signal in state-specific datasets and moderate temporal structure in the focal North American lineage (Appendix Table 4). TreeTime (https://github.com/neherlab/treetime) estimated a tMRCA of June 14, 2024 (90% CI December 24, 2023–December 12, 2024) for the expanding North American D8 measles virus lineage, supporting recent shared ancestry and expansion of a sublineage in Texas within broader North American D8 measles virus diversity. 

**Figure 3:**
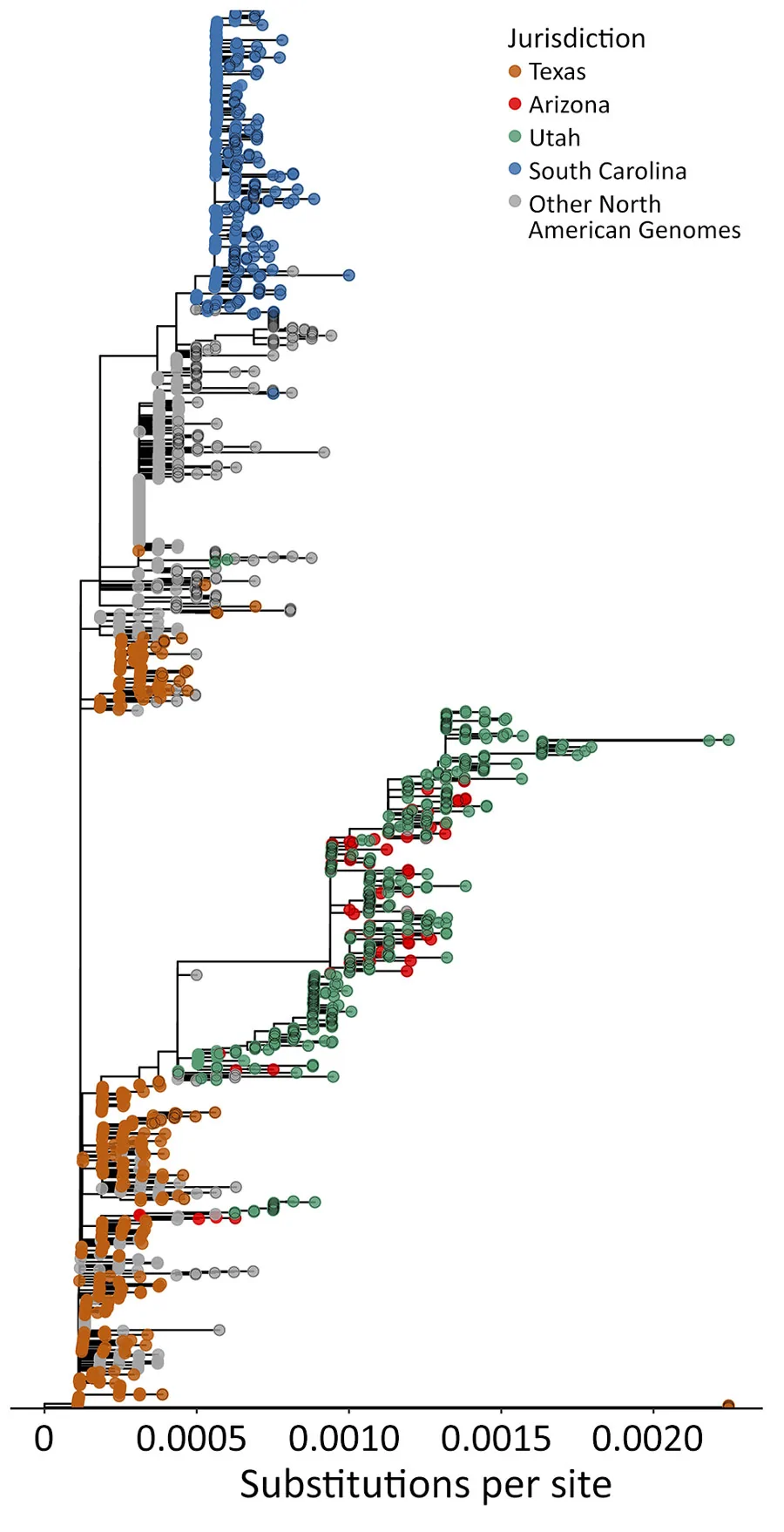
Maximum-likelihood phylogeny of the focal expanding North American genotype D8 measles virus lineage from study of whole-genome sequencing of measles virus from 2025 outbreak, Texas, USA. Phylogeny shows 1,760 genomes from Texas, Utah, Arizona, South Carolina, and other jurisdictions in North America included in the focal expanding North American genotype D8 lineage. We excluded 11 contextual genomes outside the focal lineage from this display. Tips are colored by jurisdiction; Canada genomes and US genomes outside Texas, Arizona, Utah, and South Carolina are grouped as other North American genomes. Tips identified by TreeTime (https://github.com/neherlab/treetime) as molecular-clock outliers are shown with black open circles. Branch lengths represent substitutions per site. The tree was rooted for visualization only; root placement should not be interpreted as evidence of ancestry, source, or transmission direction.

We observed lower pairwise SNP distances within Texas than between Texas and Utah or Arizona, indicating a more compact genetic cluster among measles virus genomes associated with the outbreak in Texas (Figure 4; Appendix Table 5). Measles virus genomes from Arizona and Utah were related to genomes from Texas but demonstrated heterogeneous phylogenetic placement. In contrast, measles virus genomes from South Carolina formed a distinct sublineage within broader North American D8 diversity. Those findings support local expansion of a closely related Texas lineage within wider North American D8 diversity, with distinguishable but related sublineages circulating concurrently in other jurisdictions. 

**Figure 4:**
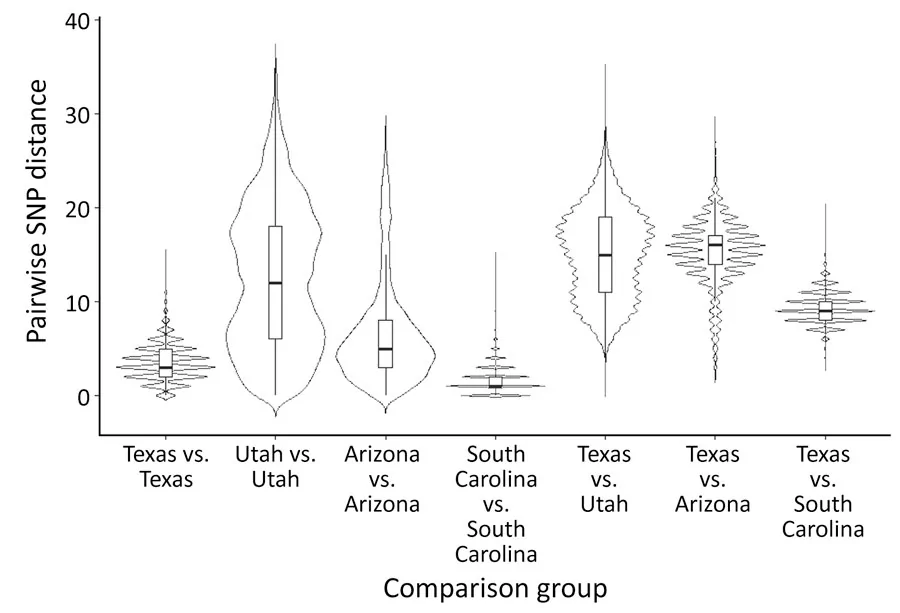
Pairwise SNP-distance distributions among genomes in the focal expanding North American genotype D8 lineage from study of whole-genome sequencing of measles virus from 2025 outbreak, Texas, USA. Violin plots show distributions of pairwise SNP distances within Texas, Utah, Arizona, and South Carolina and between Texas and each comparison jurisdiction. Embedded boxplots show medians (horizonal lines) and interquartile ranges (box tops and bottoms); whiskers indicate the most extreme values within 1.5x the interquartile range below the first quartile or above the third quartile. Wider sections of violins indicate greater density of pairwise comparisons. SNP, single-nucleotide polymorphism.

## Conclusion

Our genomic investigation of the largest after elimination measles outbreak in Texas demonstrated expansion of a closely related genotype D8 lineage and demonstrated the added resolution of WGS beyond routine N-450 genotyping. Measles virus genomes from Texas clustered closely with other D8 measles genotypes from North America, including those from Arizona, Utah, and South Carolina. However, incomplete regional sampling, limited WGS availability from contemporaneous outbreaks, variable MF-NCR coverage, low sequence diversity, and molecular-clock outliers prevented definitive inference of geographic origin or transmission direction. We therefore interpreted molecular clock estimates as temporal context rather than evidence of jurisdictional source or directionality. 

The F:H419R mutation was fixed among measles genomes from Texas and was detected among recent publicly available D8 whole genomes, supporting its use as a lineage-associated genomic surveillance marker. Because publicly available data are enriched for WGS whereas routine measles surveillance commonly relies on N-450 sequencing, our analysis cannot estimate the prevalence or geographic distribution of this mutation. 

Our findings demonstrate how WGS can distinguish closely related measles virus lineages that routine N-450 genotyping cannot resolve and complement epidemiologic investigation during large outbreaks. Expanding timely, geographically representative WGS and integrating genomic and epidemiologic data could strengthen measles surveillance. 

Appendix Additional information about whole-genome sequencing of measles virus from 2025 outbreak, Texas, USA
