# Supplementary material for: Whole-Genome Sequencing of Measles Virus from 2025 Outbreak, Texas, USA
Source: Emerg Infect Dis. 2026 Oct;32(10):1716–20. doi: 10.3201/eid3210.260494 (PMC13632840; doi:10.3201/eid3210.260494)
Supplement: Supplementary file 1 [file 26-0494-Techapp-s1.pdf]

Article DOI: https://doi.org/10.3201/eid3210.260494

EID cannot ensure accessibility for supplementary materials supplied by authors. Readers who have difficulty accessing supplementary content should contact the authors for assistance.

Whole-Genome Sequencing of Measles Virus from 2025 Outbreak, Texas, USA

Appendix

Appendix Method

Sequencing, Assembly, and Quality Assessment

RNA was reverse transcribed using the LunaScript® RT SuperMix Kit (New England Biolabs). Amplicon-based sequencing followed a modified ARTIC-style protocol originally developed for SARS-CoV-2 (https://dx.doi.org/10.17504/protocols.io.bfefjjbn and https://dx.doi.org/10.17504/protocols.io.bffyjjpw), adapted with two primer pools designed for measles virus (C. Kent et al., unpub. data, https://doi.org/10.1101/2024.12.20.629611). PCR amplification used Q5® Hot Start High-Fidelity DNA Polymerase (New England Biolabs). Amplicons were purified with AMPure XP magnetic beads (Beckman Coulter), and sequencing libraries were prepared with the Illumina DNA Prep kit (Illumina). Libraries were quantified, normalized, and sequenced on the Illumina MiSeq platform using 2 × 250 bp paired-end reads.

Raw reads were demultiplexed, adaptor-trimmed, and quality-filtered. Consensus genome assembly was performed using Cecret pipeline v3.15.24191 (https://github.com/UPHL-BioNGS/Cecret). The ARTIC/PrimalScheme measles 400 bp v1.0.0 (https://labs.primalscheme.com/detail/artic-measles/400/v1.0.0/?q%20=) primer BED and reference FASTA use NC_001498.1 (Ichinose-B95a) as the coordinate reference; therefore, NC_001498.1 was used for read mapping, primer-coordinate consistency, primer trimming, and consensus generation. NC_001498.1 was not used as a contemporary outbreak comparator for phylogenetic interpretation.

To address reference choice and coverage comparability, we performed a post-consensus D8-reference-based reassessment. Final consensus genomes were aligned with the complete genotype D8 reference genome KJ018971.2 (*1*) using MAFFT v7.526 (*2*), and callable coverage

was recalculated relative to D8 reference-homologous positions. Insertion columns introduced during multiple-sequence alignment were excluded from coverage calculations. Genomes with ≥80% D8 reference-based callable coverage were retained for phylogenetic analysis. Among Texas genomes, median reference-based callable coverage was 91.2% (interquartile range [IQR] 88.8%–94.1%; range 80.1%–99.2%).

We also quantified shared callable sites across the North American alignment. For the North American analysis, we combined the 368 Texas outbreak genomes generated in this study with publicly available measles virus whole-genome sequences with collection dates from 2024 through 2026–04–29, including genomes from Arizona (n = 104), South Carolina (n = 369), Utah (n = 371), other U.S. states (n = 100), and Canada (n = 2) obtained from the NCBI Virus database on 2026–04–29. Additional Canadian genomes from Pathoplexus (n = 457) were included as background contextual sequences under Pathoplexus data-use terms (SeqSet DOI: https://doi.org/10.62599/PP_SS_1486.5). These Canadian genomes were used to improve North American genotype D8 context and were not analyzed as a separate outbreak group or used to infer Canadian outbreak origin or transmission direction. The final North American contextual dataset included 1,771 genomes.

Across 1,771 genomes, 81.2% of KJ018971.2 reference positions were callable in ≥95% of genomes, 84.9% were callable in ≥90% of genomes, and 91.8% were callable in ≥80% of genomes. Region-specific callable coverage was calculated by mapping KJ018971.2 reference coordinates to alignment columns and summarizing coverage across measles virus genomic regions. Coverage was high across most coding regions, including N-450 and the Fusion gene, but was more variable in the matrix–fusion noncoding region (MF-NCR). Because MF-NCR coverage differed across datasets, we repeated phylogenetic reconstruction after masking MF-NCR positions. The major clustering pattern was retained after MF-NCR masking; therefore, broad lineage relationships were considered robust.

Consensus alignments were visually inspected to assess homologous placement of coding regions and to identify gap-rich or poorly covered regions. No obvious sample-specific misalignment was observed across coding regions used for phylogenetic analysis. A prominent gap-rich region overlapped the matrix–fusion noncoding region (MF-NCR), consistent with variable coverage in this region. Two genomes in the North American dataset, TXMEV2500094

and PZ168480.1, showed localized abnormal gap/alignment structure in the MF-NCR-associated region during manual inspection and were also identified by TreeTime as molecular-clock outliers (see “North American Comparative Phylogenetic Analysis” for TreeTime analysis). These genomes were retained for QC documentation but were not used to support clock-based temporal interpretation or fine-scale pairwise SNP interpretation.

Phylogenetic Analysis of Texas Measles Outbreak Samples

Consensus genomes for the Texas measles outbreak (n = 368) were aligned with the genotype D8 reference genome KJ018971.2 using MAFFT v7.526. This alignment, which included KJ018971.2 for reference placement and visualization, was used to infer the maximum-likelihood phylogeny shown in Figure 1A. IQ-TREE v2.4.0 (*3*) was used for phylogenetic inference. The best-fit model of sequence evolution for this Texas-plus-reference alignment was TIM+F+G4, selected by ModelFinder based on AIC (*4*). Node support was estimated by ultrafast bootstrap (*5*) with 1,000 replicates. Collection dates and patient county were used to annotate the phylogenetic tree.

TMRCA Estimation for the Texas-Only Dataset

For molecular-clock analysis, the temporally distant D8 reference genome KJ018971.2 was excluded. The Texas-only alignment (n = 368) was analyzed separately for temporal signal and exploratory TMRCA estimation. For the Texas-only TreeTime input tree, ModelFinder selected TIM+F+I+R6 based on AIC. TreeTime v0.11.4 (*6*) was used for TMRCA estimation with the following command:

treetime–tree tree.nhx–aln alignment.fasta–dates timeline.tsv–outdir treetime_out–confidence–covariation–stochastic-resolve–relax 0.001 0.0001

We used TreeTime’s relaxed-clock option (–relax 0.001 0.0001) to allow limited branch-specific rate variation and–covariation to account for covariance among related samples during date estimation. Node-date confidence intervals were estimated with–confidence, and polytomies were resolved with–stochastic-resolve.

Root-to-tip regression of genetic distance against sampling date was used to assess temporal signal in the Texas-only dataset (Appendix Figure 1). Because the Texas-only dataset showed limited temporal signal, the Texas-only TMRCA was treated as exploratory and was not used to infer introduction timing.

Genome-Wide Association Study

We used Pyseer v1.4.0 to conduct a phylogeny-based linear mixed-model genome-wide association study (GWAS) to identify SNPs associated with patient vaccination status (*7*). The variants were detected by aligning trimmed paired reads of the 368 sequences to a reference D8 genome (KJ018971.2) via the software Snippy v4.6.0. The resulting core SNP alignment was used to generate an approximately maximum-likelihood phylogenetic tree with FastTree (2.1.11) (*8*), which we then input into the GWAS model to control population structure.

Only core variants with allele frequency >1% were tested. For the primary vaccination-status GWAS excluding unknown-status cases, 5 variants passed filtering and no variant met the Bonferroni-corrected significance threshold of 1.00×10^{-2}. F:H419R could not be evaluated as a predictor of vaccination status because it was fixed among Texas genomes. In a sensitivity analysis grouping unknown-status and unvaccinated cases together, 11 variants passed filtering and no variant met the Bonferroni-corrected significance threshold of 4.55×10^{-3}.

Selection Inference Analysis

To identify codons under selection in our D8 dataset, we began by assembling trimmed reads with Unicycler v0.4.8 (*9*) and annotating the genomes with Prokka v 1.14.6 (*10*). Only full-length sequences were considered for further analysis (fusion protein, n = 349; hemagglutinin protein, n = 362; matrix protein, n = 357; nucleoprotein, n = 210; phosphoprotein, n = 352; polymerase, n = 337). We used FastTree to construct approximately maximum-likelihood phylogenetic trees from the coding sequences of each protein. The phylogenetic tree, along with the corresponding protein-coding sequence alignment, was used to run FUBAR (Fast, Unconstrained Bayesian AppRoximation) to test for pervasive site-level selection (*11*). The FUBAR test was conducted via the HyPhy software v2.5.9. Our posterior probability cutoff was 0.9.

Global Context Placement of the Texas outbreak

To evaluate the distribution of the Fusion protein substitution F:H419R, publicly available genotype D8 genomes with collection dates from 2023–01–01 through 2025–09–24 were downloaded from the NCBI Virus database. F:H419R status was determined from consensus FASTA sequences by translating the Fusion coding sequence. Genomes were classified only when the codon encoding Fusion residue 419 contained unambiguous nucleotide

calls; genomes with Ns, gaps, or ambiguous bases at that codon were excluded from F:H419R analysis. Frequencies were summarized by collection year. MIBE-associated sequences and sequences without sufficient Fusion gene coverage were excluded from F:H419R frequency summaries.

To place the Texas outbreak genomes within broader global genotype D8 diversity, we used the Nextstrain measles workflow (https://github.com/nextstrain/measles) to construct a contextual phylogeny. Nearly complete publicly available measles virus genomes were downloaded from the NCBI Virus database on 2025–09–24. Genomes shorter than 12.5 kb were excluded. Texas outbreak genomes generated in this study were combined with the global reference dataset for phylogenetic analysis.

Sequences were aligned using the default Nextstrain measles workflow parameters. Time-resolved phylogenetic reconstruction was performed using IQ-TREE and TreeTime as implemented in the workflow. To reduce geographic and temporal sampling imbalance while preserving broad global representation, publicly available genomes were subsampled to a maximum of 30 genomes per country–year group. Consequently, the displayed phylogeny represents a contextual subset of available global genomes rather than all publicly available sequences.

North American Comparative Phylogenetic Analysis

For the North American comparative analysis, we assembled a contextual dataset of 1,771 measles virus whole genomes from Texas, Arizona, Utah, South Carolina, Canada, and other North American jurisdictions (see “Sequencing, Assembly, and Quality Assessment” for dataset description). This dataset was used for broad phylogenetic placement and molecular-clock assessment. After phylogenetic reconstruction, 11 genomes fell outside the focal expanding North American genotype D8 lineage and were retained only as contextual background: PP_004J0LL.1, PP_004J0MJ.1, PQ873025.1, PQ873024.1, PP_006JYQC.1, PX437932.1, PZ017002.1, PZ194287.1, PZ194288.1, PZ194289.1, and PZ259696.1. The remaining 1,760 genomes comprised the focal expanding North American lineage used for lineage-specific pairwise SNP-distance comparisons and root-to-tip regression diagnostics. This distinction avoided conflating background D8 diversity with the outbreak-associated North American lineage.

Sequence alignment was performed with MAFFT v7.526. A maximum-likelihood tree was inferred with IQ-TREE v2.4.0 using 1,000 ultrafast bootstrap replicates. The best-fit model of sequence evolution, TIM+F+I+R6, was selected by ModelFinder based on AIC.

Time-scaled phylogenetic inference was performed with TreeTime v0.11.4 using sampling dates and the same relaxed-clock model and parameters described above. Clock-filtered tips were interpreted as genomes with poor temporal fit under the fitted molecular-clock model and were not used to infer transmission direction. TXMEV2500094 and PZ168480.1 were among the TreeTime clock-filtered tips and also showed localized abnormal alignment/gap structure in the MF-NCR-associated region. These genomes were therefore treated as QC-flagged outliers for North American temporal analyses and fine-scale pairwise interpretation.

Temporal signal was assessed separately with Clockor2 (*12*) under strict-clock root-to-tip regression using the same maximum-likelihood trees used for TreeTime analysis. For each dataset, root-to-tip genetic distance was regressed against sample collection date to evaluate clock-like accumulation of sequence divergence. The best-fitting root was selected by maximizing R^2. Estimated substitution rate, R^2, residual mean squared error, and intercept were recorded as molecular-clock diagnostics (Appendix Table 4). Because Clockor2 evaluates strict-clock root-to-tip regression whereas TreeTime TMRCA estimation used a relaxed-clock model with clock-filtered tips excluded from rate fitting, substitution-rate estimates from the two approaches were not directly compared. Instead, Clockor2 results were used as diagnostic evidence of temporal signal before interpreting TreeTime estimates.

Pairwise SNP Distance Calculation

Pairwise SNP distances were calculated from the whole-genome consensus alignment by using mutually callable sites only. For each genome pair, nucleotide differences were counted only at positions where both genomes had unambiguous A/C/G/T calls; positions containing Ns, gaps, or ambiguous nucleotide codes in either genome were excluded. Because callable regions differed among genomes, pairwise comparisons were filtered at the pair level: pairs with <80% shared callable overlap were excluded from distance summaries, even if both genomes met the overall genome-level inclusion threshold.

Pairwise SNP-distance analyses were restricted to genomes within the focal expanding North American D8 lineage. Genomes outside this lineage were retained for phylogenetic

context but excluded from lineage-specific pairwise comparisons to avoid conflating background D8 diversity with the outbreak-associated lineage. Comparisons involving TXMEV2500094 and PZ168480.1 were also excluded from pairwise SNP-distance visualization and summary because both genomes showed localized abnormal alignment/gap structure in the MF-NCR-associated region and were independently identified by TreeTime as molecular-clock outliers.

References

1. Gardy JL, Naus M, Amlani A, Chung W, Kim H, Tan M, et al. Whole-genome sequencing of measles virus genotypes H1 and D8 during outbreaks of infection following the 2010 Olympic Winter Games reveals viral transmission routes. J Infect Dis. 2015;212:1574–8. PubMed https://doi.org/10.1093/infdis/jiv271

2. Katoh K, Standley DM. MAFFT multiple sequence alignment software version 7: improvements in performance and usability. Mol Biol Evol. 2013;30:772–80. PubMed https://doi.org/10.1093/molbev/mst010

3. Minh BQ, Schmidt HA, Chernomor O, Schrempf D, Woodhams MD, von Haeseler A, et al. IQ-TREE 2: new models and efficient methods for phylogenetic inference in the genomic era. Mol Biol Evol. 2020;37:1530–4. PubMed https://doi.org/10.1093/molbev/msaa015

4. Kalyaanamoorthy S, Minh BQ, Wong TKF, von Haeseler A, Jermiin LS. ModelFinder: fast model selection for accurate phylogenetic estimates. Nat Methods. 2017;14:587–9. PubMed https://doi.org/10.1038/nmeth.4285

5. Hoang DT, Chernomor O, von Haeseler A, Minh BQ, Vinh LS. UFBoot2: improving the ultrafast bootstrap approximation. Mol Biol Evol. 2018;35:518–22. PubMed https://doi.org/10.1093/molbev/msx281

6. Sagulenko P, Puller V, Neher RA. TreeTime: maximum-likelihood phylodynamic analysis. Virus Evol. 2018;4:vex042. PubMed https://doi.org/10.1093/ve/vex042

7. Lees JA, Galardini M, Bentley SD, Weiser JN, Corander J. pyseer: a comprehensive tool for microbial pangenome-wide association studies. Bioinformatics. 2018;34:4310–2. PubMed https://doi.org/10.1093/bioinformatics/bty539

8. Price MN, Dehal PS, Arkin AP. FastTree 2—approximately maximum-likelihood trees for large alignments. PLoS One. 2010;5:e9490. PubMed https://doi.org/10.1371/journal.pone.0009490

9. Wick RR, Judd LM, Gorrie CL, Holt KE. Unicycler: resolving bacterial genome assemblies from short and long sequencing reads. PLOS Comput Biol. 2017;13:e1005595. PubMed https://doi.org/10.1371/journal.pcbi.1005595

10. Seemann T. Prokka: rapid prokaryotic genome annotation. Bioinformatics. 2014;30:2068–9. PubMed https://doi.org/10.1093/bioinformatics/btu153

11. Murrell B, Moola S, Mabona A, Weighill T, Sheward D, Kosakovsky Pond SL, et al. FUBAR: a fast, unconstrained bayesian approximation for inferring selection. Mol Biol Evol. 2013;30:1196–205. PubMed https://doi.org/10.1093/molbev/mst030

12. Featherstone LA, Rambaut A, Duchene S, Wirth W. Clockor2: inferring global and local strict molecular clocks using root-to-tip regression. Syst Biol. 2024;73:623–8. PubMed https://doi.org/10.1093/sysbio/syae003

Appendix Table 1. Texas measles outbreak sample list. WHO-format strain names were assigned for NCBI submission using standard measles virus nomenclature. CDC-assigned strain names are indicated where available. N-450 distinct sequence identifiers were reported only when assigned or available; otherwise, entries are listed as not available because the authors did not have direct access to the MeaNS DSId assignment database.

Index	ID	Alternative ID	CDC/WHO Strain name	clade	DSId	date
1	TXMEV2500001	AVI2500841	MVs/Texas.USA/6.25/7	D8	9171	2025–02–06
2	TXMEV2500006	AVI2501210	MVs/Texas.USA/9.25/4	D8	not available	2025–02–24
3	TXMEV2500008	AVI2500744	MVs/Texas.USA/4.25/3	D8	9171	2025–01–30
4	TXMEV2500009	AVI2500777	MVs/Texas.USA/6.25	D8	9171	2025–02–04
5	TXMEV2500010	AVI2500837	MVs/Texas.USA/6.25/5	D8	9171	2025–02–06
6	TXMEV2500011	AVI2500843	MVs/Texas.USA/5.25/2	D8	9171	2025–02–01
7	TXMEV2500012	AVI2500870	MVs/Texas.USA/6.25/8	D8	9171	2025–02–07
8	TXMEV2500013	AVI2500925	MVs/Texas.USA/7.25/2	D8	9171	2025–02–11
9	TXMEV2500014	AVI2500950	MVs/Texas.USA/7.25/9	D8	9171	2025–02–12
10	TXMEV2500015	AVI2501043	MVs/Texas.USA/7.25/14	D8	9171	2025–02–17
11	TXMEV2500016	AVI2501066	MVs/Texas.USA/7.25/16	D8	9171	2025–02–15
12	TXMEV2500017	AVI2501068	MVs/Texas.USA/8.25/4	D8	9171	2025–02–16
13	TXMEV2500018	AVI2501093	MVs/Texas.USA/8.25/13	D8	9171	2025–02–18
14	TXMEV2500021	AVI2501373	MVs/Texas.USA/9.25/9	D8	9171	2025–03–04
15	TXMEV2500023	AVI2500779	MVs/Texas.USA/5.25	D8	9171	2025–02–03
16	TXMEV2500025	AVI2501098	MVs/Texas.USA/8.25/18	D8	9171	2025–02–18
17	TXMEV2500027	AVI2500778	MVs/Texas.USA/6.25/2	D8	9171	2025–02–04
18	TXMEV2500028	AVI2500780	MVs/Texas.USA/6.25/3	D8	9171	2025–02–03
19	TXMEV2500029	AVI2500836	MVs/Texas.USA/6.25/4	D8	9171	2025–02–06
20	TXMEV2500030	AVI2500851	MVs/Texas.USA/6.25/6	D8	9171	2025–02–07
21	TXMEV2500031	AVI2500871	MVs/Texas.USA/6.25/9	D8	9171	2025–02–06
22	TXMEV2500032	AVI2500922	MVs/Texas.USA/5.25/3	D8	9171	2025–02–11
23	TXMEV2500033	AVI2500931	MVs/Texas.USA/7.25/3	D8	9171	2025–02–11
24	TXMEV2500034	AVI2500941	MVs/Texas.USA/7.25/4	D8	9171	2025–02–11
25	TXMEV2500035	AVI2500942	MVs/Texas.USA/7.25/5	D8	9171	2025–02–11
26	TXMEV2500036	AVI2500945	MVs/Texas.USA/7.25/6	D8	9171	2025–02–11
27	TXMEV2500037	AVI2500946	MVs/Texas.USA/7.25/7	D8	9171	2025–02–11
28	TXMEV2500039	AVI2500949	MVs/Texas.USA/7.25/8	D8	9171	2025–02–12
29	TXMEV2500040	AVI2500952	MVs/Texas.USA/7.25/10	D8	9171	2025–02–11
30	TXMEV2500041	AVI2500956	MVs/Texas.USA/7.25/11	D8	9171	2025–02–12
31	TXMEV2500042	AVI2500970	MVs/Texas.USA/7.25/12	D8	not available	2025–02–12
32	TXMEV2500043	AVI2500971	MVs/Texas.USA/6.25/10	D8	9171	2025–02–12
33	TXMEV2500044	AVI2500980	MVs/Texas.USA/7.25/14	D8	not available	2025–02–12
34	TXMEV2500045	AVI2500993	MVs/Texas.USA/7.25/15	D8	not available	2025–02–14
35	TXMEV2500046	AVI2501039	MVs/Texas.USA/7.25/12	D8	9171	2025–02–16
36	TXMEV2500047	AVI2501040	MVs/Texas.USA/7.25/13	D8	9171	2025–02–16
37	TXMEV2500048	AVI2501045	MVs/Texas.USA/8.25/2	D8	9171	2025–02–17
38	TXMEV2500049	AVI2501053	MVs/Texas.USA/8.25/3	D8	9171	2025–02–17

Index	ID	Alternative ID	CDC/WHO Strain name	clade	DSId	date
39	TXMEV2500050	AVI2501069	MVs/Texas.USA/8.25/5	D8	9171	2025–02–17
40	TXMEV2500051	AVI2501070	MVs/Texas.USA/7.25/15	D8	9171	2025–02–15
41	TXMEV2500052	AVI2501074	MVs/Texas.USA/8.25/7	D8	9171	2025–02–16
42	TXMEV2500053	AVI2501084	MVs/Texas.USA/8.25/9	D8	9171	2025–02–18
43	TXMEV2500054	AVI2501085	MVs/Texas.USA/8.25/10	D8	9171	2025–02–18
44	TXMEV2500055	AVI2501088	MVs/Texas.USA/8.25/11	D8	9171	2025–02–20
45	TXMEV2500056	AVI2501092	MVs/Texas.USA/8.25/12	D8	9171	2025–02–18
46	TXMEV2500057	AVI2501094	MVs/Texas.USA/8.25/14	D8	9171	2025–02–19
47	TXMEV2500058	AVI2501095	MVs/Texas.USA/8.25/15	D8	9171	2025–02–19
48	TXMEV2500059	AVI2501097	MVs/Texas.USA/8.25/17	D8	9171	2025–02–20
49	TXMEV2500060	AVI2501099	MVs/Texas.USA/8.25/19	D8	9171	2025–02–18
50	TXMEV2500061	AVI2501101	MVs/Texas.USA/8.25/18	D8	not available	2025–02–19
51	TXMEV2500062	AVI2501109	MVs/Texas.USA/8.25/21	D8	9171	2025–02–18
52	TXMEV2500063	AVI2501110	MVs/Texas.USA/8.25/22	D8	9171	2025–02–19
53	TXMEV2500064	AVI2501112	MVs/Texas.USA/8.25/23	D8	9171	2025–02–20
54	TXMEV2500065	AVI2501115	MVs/Texas.USA/8.25/24	D8	9171	2025–02–19
55	TXMEV2500066	AVI2501117	MVs/Texas.USA/8.25/25	D8	9171	2025–02–20
56	TXMEV2500067	AVI2501118	MVs/Texas.USA/8.25/26	D8	9171	2025–02–20
57	TXMEV2500068	AVI2501145	MVs/Texas.USA/7.25/17	D8	9171	2025–02–24
58	TXMEV2500069	AVI2500573	MVs/Texas.USA/4.25/2	D8	9171	2025–01–27
59	TXMEV2500070	AVI2500667	MVs/Texas.USA/4.25	D8	9171	2025–01–28
60	TXMEV2500073	AVI2501096	MVs/Texas.USA/8.25/16	D8	9171	2025–02–19
61	TXMEV2500075	AVI2501149	MVs/Texas.USA/9.25	D8	9171	2025–02–23
62	TXMEV2500076	AVI2501150	MVs/Texas.USA/8.25/27	D8	9171	2025–02–21
63	TXMEV2500077	AVI2501151	MVs/Texas.USA/8.25/20	D8	9171	2025–02–22
64	TXMEV2500078	AVI2501155	MVs/Texas.USA/8.25/28	D8	9171	2025–02–21
65	TXMEV2500079	AVI2501156	MVs/Texas.USA/8.25/32	D8	9171	2025–02–21
66	TXMEV2500080	AVI2501157	MVs/Texas.USA/8.25/31	D8	9274	2025–02–20
67	TXMEV2500081	AVI2501158	MVs/Texas.USA/8.25/29	D8	9274	2025–02–20
68	TXMEV2500082	AVI2501160	MVs/Texas.USA/8.25/30	D8	9274	2025–02–20
69	TXMEV2500083	AVI2501161	MVs/Texas.USA/8.25/41	D8	not available	2025–02–20
70	TXMEV2500085	AVI2501163	MVs/Texas.USA/8.25/34	D8	not available	2025–02–21
71	TXMEV2500086	AVI2501166	MVs/Texas.USA/8.25/38	D8	not available	2025–02–22
72	TXMEV2500087	AVI2501167	MVs/Texas.USA/8.25/35	D8	not available	2025–02–21
73	TXMEV2500088	AVI2501168	MVs/Texas.USA/8.25/39	D8	not available	2025–02–22
74	TXMEV2500089	AVI2501169	MVs/Texas.USA/8.25/40	D8	not available	2025–02–22
75	TXMEV2500090	AVI2501170	MVs/Texas.USA/9.25/2	D8	not available	2025–02–23
76	TXMEV2500091	AVI2501171	MVs/Texas.USA/8.25/36	D8	not available	2025–02–21
77	TXMEV2500092	AVI2501178	MVs/Texas.USA/9.25/3	D8	not available	2025–02–23
78	TXMEV2500093	AVI2501215	MVs/Texas.USA/9.25/9	D8	not available	2025–02–25
79	TXMEV2500094	AVI2501220	MVs/Texas.USA/9.25/10	D8	not available	2025–02–25
80	TXMEV2500095	AVI2501221	MVs/Texas.USA/9.25/6	D8	not available	2025–02–24
81	TXMEV2500096	AVI2501223	MVs/Texas.USA/9.25/11	D8	not available	2025–02–25
82	TXMEV2500097	AVI2501224	MVs/Texas.USA/9.25/12	D8	not available	2025–02–25
83	TXMEV2500098	AVI2501227	MVs/Texas.USA/9.25/7	D8	not available	2025–02–24
84	TXMEV2500099	AVI2501228	MVs/Texas.USA/9.25/8	D8	not available	2025–02–24
85	TXMEV2500100	AVI2501258	MVs/Texas.USA/9.25/21	D8	not available	2025–02–27
86	TXMEV2500101	AVI2501269	MVs/Texas.USA/9.25/13	D8	not available	2025–02–26
87	TXMEV2500103	AVI2501271	MVs/Texas.USA/9.25/14	D8	not available	2025–02–26
88	TXMEV2500105	AVI2501274	MVs/Texas.USA/9.25/15	D8	not available	2025–02–26
89	TXMEV2500106	AVI2501276	MVs/Texas.USA/9.25/16	D8	not available	2025–02–26
90	TXMEV2500107	AVI2501280	MVs/Texas.USA/9.25/17	D8	not available	2025–02–26
91	TXMEV2500108	AVI2501281	MVs/Texas.USA/9.25/18	D8	not available	2025–02–26
92	TXMEV2500109	AVI2501287	MVs/Texas.USA/9.25/19	D8	not available	2025–02–26
93	TXMEV2500110	AVI2501314	MVs/Texas.USA/10.25	D8	not available	2025–03–02
94	TXMEV2500111	AVI2501315	MVs/Texas.USA/9.25/20	D8	not available	2025–02–26
95	TXMEV2500112	AVI2501324	MVs/Texas.USA/9.25/26	D8	not available	2025–02–28
96	TXMEV2500113	AVI2501325	MVs/Texas.USA/9.25/27	D8	not available	2025–02–28
97	TXMEV2500114	AVI2501326	MVs/Texas.USA/9.25/31	D8	not available	2025–03–01
98	TXMEV2500115	AVI2501327	MVs/Texas.USA/10.25/2	D8	not available	2025–03–02
99	TXMEV2500116	AVI2501328	MVs/Texas.USA/9.25/32	D8	not available	2025–03–01
100	TXMEV2500117	AVI2501329	MVs/Texas.USA/10.25/3	D8	not available	2025–03–02
101	TXMEV2500118	AVI2501330	MVs/Texas.USA/10.25/4	D8	not available	2025–03–02
102	TXMEV2500119	AVI2501331	MVs/Texas.USA/9.25/22	D8	not available	2025–02–27
103	TXMEV2500120	AVI2501337	MVs/Texas.USA/9.25/10	D8	9171	2025–03–03
104	TXMEV2500121	AVI2501354	MVs/Texas.USA/10.25/5	D8	not available	2025–03–02
105	TXMEV2500122	AVI2501368	MVs/Texas.USA/10.25/14	D8	not available	2025–03–05
106	TXMEV2500123	AVI2501370	MVs/Texas.USA/10.25/7	D8	not available	2025–03–03
107	TXMEV2500124	AVI2501371	MVs/Texas.USA/10.25/9	D8	not available	2025–03–04

Index	ID	Alternative ID	CDC/WHO Strain name	clade	DSId	date
108	TXMEV2500125	AVI2501372	MVs/Texas.USA/10.25/10	D8	not available	2025–03–04
109	TXMEV2500126	AVI2501375	MVs/Texas.USA/10.25/11	D8	not available	2025–03–04
110	TXMEV2500127	AVI2501383	MVs/Texas.USA/10.25/15	D8	not available	2025–03–05
111	TXMEV2500128	AVI2501384	MVs/Texas.USA/10.25/16	D8	not available	2025–03–05
112	TXMEV2500129	AVI2501385	MVs/Texas.USA/10.25/17	D8	not available	2025–03–05
113	TXMEV2500130	AVI2501392	MVs/Texas.USA/10.25/23	D8	not available	2025–03–06
114	TXMEV2500131	AVI2501397	MVs/Texas.USA/10.25/31	D8	not available	2025–03–08
115	TXMEV2500132	AVI2501400	MVs/Texas.USA/10.25/27	D8	not available	2025–03–07
116	TXMEV2500133	AVI2501401	MVs/Texas.USA/10.25/28	D8	not available	2025–03–07
117	TXMEV2500134	AVI2501402	MVs/Texas.USA/10.25/29	D8	not available	2025–03–07
118	TXMEV2500135	AVI2501404	MVs/Texas.USA/11.25	D8	not available	2025–03–09
119	TXMEV2500136	AVI2501405	MVs/Texas.USA/10.25/30	D8	not available	2025–03–07
120	TXMEV2500137	AVI2501406	MVs/Texas.USA/10.25/24	D8	not available	2025–03–06
121	TXMEV2500138	AVI2501407	MVs/Texas.USA/10.25/25	D8	not available	2025–03–06
122	TXMEV2500140	AVI2501416	MVs/Texas.USA/11.25/28	D8	not available	2025–03–10
123	TXMEV2500141	AVI2501422	MVs/Texas.USA/Mar.25/1790	D8	9171	2025–03–10
124	TXMEV2500142	AVI2501423	MVs/Texas.USA/Mar.25/1473	D8	9171	2025–03–10
125	TXMEV2500143	AVI2501424	MVs/Texas.USA/11.25/5	D8	not available	2025–03–10
126	TXMEV2500144	AVI2501425	MVs/Texas.USA/11.25/21	D8	not available	2025–03–12
127	TXMEV2500145	AVI2501426	MVs/Texas.USA/11.25/14	D8	not available	2025–03–11
128	TXMEV2500146	AVI2501427	MVs/Texas.USA/11.25/15	D8	not available	2025–03–11
129	TXMEV2500147	AVI2501428	MVs/Texas.USA/11.25/16	D8	not available	2025–03–11
130	TXMEV2500148	AVI2501429	MVs/Texas.USA/11.25/17	D8	not available	2025–03–11
131	TXMEV2500149	AVI2501431	MVs/Texas.USA/11.25/6	D8	not available	2025–03–10
132	TXMEV2500150	AVI2501444	MVs/Texas.USA/Mar.25/1541	D8	9171	2025–03–11
133	TXMEV2500151	AVI2501445	MVs/Texas.USA/Mar.25/1508	D8	9171	2025–03–11
134	TXMEV2500152	AVI2501482	MVs/Texas.USA/12.25/27	D8	not available	2025–03–19
135	TXMEV2500153	AVI2501483	MVs/Texas.USA/12.25/28	D8	not available	2025–03–19
136	TXMEV2500154	AVI2501484	MVs/Texas.USA/12.25/29	D8	not available	2025–03–19
137	TXMEV2500155	AVI2501487	MVs/Texas.USA/Mar.25/1384	D8	9171	2025–03–17
138	TXMEV2500156	AVI2501488	MVs/Texas.USA/12.25/19	D8	not available	2025–03–18
139	TXMEV2500157	AVI2501492	MVs/Texas.USA/12.25/39	D8	not available	2025–03–21
140	TXMEV2500158	AVI2501494	MVs/Texas.USA/12.25/40	D8	not available	2025–03–21
141	TXMEV2500159	AVI2501496	MVs/Texas.USA/12.25/32	D8	not available	2025–03–20
142	TXMEV2500160	AVI2501497	MVs/Texas.USA/12.25/33	D8	not available	2025–03–20
143	TXMEV2500165	AVI2501453	MVs/Texas.USA/Mar.25/1242	D8	9171	2025–03–16
144	TXMEV2500166	AVI2501454	MVs/Texas.USA/12.25/6	D8	not available	2025–03–17
145	TXMEV2500169	AVI2501490	MVs/Texas.USA/12.25/20	D8	not available	2025–03–18
146	TXMEV2500170	AVI2501491	MVs/Texas.USA/12.25/21	D8	not available	2025–03–18
147	TXMEV2500171	AVI2501498	MVs/Texas.USA/12.25/34	D8	not available	2025–03–20
148	TXMEV2500174	AVI2501519	MVs/Texas.USA/13.25/7	D8	not available	2025–03–24
149	TXMEV2500175	AVI2501524	MVs/Texas.USA/13.25/13	D8	not available	2025–03–25
150	TXMEV2500176	AVI2501525	MVs/Texas.USA/13.25/18	D8	not available	2025–03–26
151	TXMEV2500177	AVI2501537	MVs/Texas.USA/14.25/16	D8	not available	2025–04–01
152	TXMEV2500181	AVI2501542	MVs/Texas.USA/13.25/23	D8	not available	2025–03–27
153	TXMEV2500182	AVI2501558	MVs/Texas.USA/Apr.25/1150	D8	9171	2025–04–01
154	TXMEV2500184	AVI2501567	MVs/Texas.USA/14.25/30	D8	not available	2025–04–05
155	TXMEV2500185	AVI2501569	MVs/Texas.USA/14.25/26	D8	not available	2025–04–04
156	TXMEV2500187	AVI2501572	MVs/Texas.USA/15.25/3	D8	not available	2025–04–07
157	TXMEV2500188	AVI2501573	MVs/Texas.USA/15.25/4	D8	not available	2025–04–07
158	TXMEV2500191	AVI2501576	MVs/Texas.USA/15.25	D8	not available	2025–04–06
159	TXMEV2500192	AVI2501584	MVs/Texas.USA/Apr.25/1679	D8	9171	2025–04–09
160	TXMEV2500193	AVI2501585	MVs/Texas.USA/Apr.25/1488	D8	9171	2025–04–09
161	TXMEV2500194	AVI2501586	MVs/Texas.USA/Apr.25/1801	D8	9171	2025–04–09
162	TXMEV2500195	AVI2501588	MVs/Texas.USA/Apr.25/1086	D8	9171	2025–04–09
163	TXMEV2500196	AVI2501590	MVs/Texas.USA/Apr.25/1387	D8	9171	2025–04–08
164	TXMEV2500197	AVI2501599	MVs/Texas.USA/15.25/10	D8	not available	2025–04–08
165	TXMEV2500200	AVI2501609	MVs/Texas.USA/Apr.25/1456	D8	9171	2025–04–12
166	TXMEV2500201	AVI2501610	MVs/Texas.USA/15.25/18	D8	not available	2025–04–10
167	TXMEV2500204	AVI2501622	MVs/Texas.USA/Apr.25/1777	D8	9171	2025–04–16
168	TXMEV2500205	AVI2501628	MVs/Texas.USA/15.25/19	D8	not available	2025–04–10
169	TXMEV2500206	AVI2501630	MVs/Texas.USA/16.25/9	D8	not available	2025–04–17
170	TXMEV2500207	AVI2501636	MVs/Texas.USA/Apr.25/1191	D8	9171	2025–04–19
171	TXMEV2500208	AVI2501637	MVs/Texas.USA/Apr.25/1550	D8	9171	2025–04–19
172	TXMEV2500209	AVI2501638	MVs/Texas.USA/Apr.25/1379	D8	9171	2025–04–19
173	TXMEV2500212	AVI2501647	MVs/Texas.USA/16.25/10	D8	not available	2025–04–17
174	TXMEV2500213	AVI2501658	MVs/Texas.USA/17.25/3	D8	not available	2025–04–22
175	TXMEV2500215	AVI2501685	MVs/Texas.USA/Apr.25/1492	D8	9171	2025–04–29
176	TXMEV2500216	AVI2501695	MVs/Texas.USA/May.25/1316	D8	9171	2025–05–01

Index	ID	Alternative ID	CDC/WHO Strain name	clade	DSId	date
177	TXMEV2500217	AVI2501699	MVs/Texas.USA/May.25/1634	D8	9171	2025–05–01
178	TXMEV2500218	AVI2501705	MVs/Texas.USA/May.25/1007	D8	9171	2025–05–04
179	TXMEV2500219	AVI2501711	MVs/Texas.USA/May.25/1534	D8	9171	2025–05–07
180	TXMEV2500220	KVI2500728	MVs/Texas.USA/10.25	D8	9171	2025–03–01
181	TXMEV2500221	KVI2500729	MVs/Texas.USA/9.25/3	D8	9171	2025–02–27
182	TXMEV2500222	KVI2500730	MVs/Texas.USA/10.25/2	D8	9171	2025–02–28
183	TXMEV2500223	KVI2500732	MVs/Texas.USA/10.25/3	D8	9171	2025–03–01
184	TXMEV2500224	KVI2500733	MVs/Texas.USA/9.25/4	D8	9171	2025–02–28
185	TXMEV2500225	KVI2500734	MVs/Texas.USA/9.25/5	D8	9171	2025–02–27
186	TXMEV2500226	KVI2500736	MVs/Texas.USA/9.25/6	D8	9171	2025–02–27
187	TXMEV2500228	KVI2500744	MVs/Texas.USA/9.25/8	D8	9171	2025–02–28
188	TXMEV2500230	KVI2500786	MVs/Texas.USA/9.25/11	D8	9171	2025–03–04
189	TXMEV2500231	KVI2500788	MVs/Texas.USA/10.25/4	D8	9171	2025–03–04
190	TXMEV2500232	KVI2500812	MVs/Texas.USA/10.25/5	D8	9171	2025–03–05
191	TXMEV2500234	KVI2500817	MVs/Texas.USA/9.25/12	D8	9171	2025–03–05
192	TXMEV2500235	KVI2500818	MVs/Texas.USA/Mar.25/1104	D8	9171	2025–03–05
193	TXMEV2500237	KVI2500826	MVs/Texas.USA/10.25/7	D8	9171	2025–03–05
194	TXMEV2500238	KVI2500827	MVs/Texas.USA/10.25/8	D8	9171	2025–03–05
195	TXMEV2500239	KVI2500828	MVs/Texas.USA/10.25/9	D8	9171	2025–03–06
196	TXMEV2500244	KVI2500839	MVs/Texas.USA/10.25/12	D8	9171	2025–03–10
197	TXMEV2500245	KVI2500841	MVs/Texas.USA/10.25/13	D8	9171	2025–03–10
198	TXMEV2500247	KVI2500845	MVs/Texas.USA/10.25/14	D8	9171	2025–03–10
199	TXMEV2500248	KVI2500855	MVs/Texas.USA/11.25/2	D8	9171	2025–03–10
200	TXMEV2500249	KVI2500857	MVs/Texas.USA/11.25/3	D8	9171	2025–03–10
201	TXMEV2500250	KVI2500858	MVs/Texas.USA/10.25/15	D8	9171	2025–03–10
202	TXMEV2500254	KVI2500878	MVs/Texas.USA/Mar.25/1785	D8	9171	2025–03–11
203	TXMEV2500255	KVI2500879	MVs/Texas.USA/Mar.25/1623	D8	9171	2025–03–12
204	TXMEV2500256	KVI2500880	MVs/Texas.USA/11.25/7	D8	9171	2025–03–12
205	TXMEV2500257	KVI2500882	MVs/Texas.USA/11.25/24	D8	not available	2025–03–12
206	TXMEV2500258	KVI2500897	MVs/Texas.USA/11.25/8	D8	9171	2025–03–12
207	TXMEV2500260	KVI2500902	MVs/Texas.USA/Mar.25/1781	D8	9171	2025–03–13
208	TXMEV2500261	KVI2500905	MVs/Texas.USA/Mar.25/1029	D8	9341	2025–03–14
209	TXMEV2500262	KVI2500907	MVs/Texas.USA/12.25	D8	9171	2025–03–17
210	TXMEV2500263	KVI2500909	MVs/Texas.USA/11.25/10	D8	9322	2025–03–14
211	TXMEV2500265	KVI2500911	MVs/Texas.USA/12.25/2	D8	9323	2025–03–16
212	TXMEV2500266	KVI2500912	MVs/Texas.USA/10.25/19	D8	9171	2025–03–10
213	TXMEV2500267	KVI2500914	MVs/Texas.USA/Mar.25/1043	D8	9171	2025–03–16
214	TXMEV2500268	KVI2500915	MVs/Texas.USA/11.25/11	D8	9171	2025–03–15
215	TXMEV2500269	KVI2500916	MVs/Texas.USA/11.25/12	D8	9171	2025–03–17
216	TXMEV2500270	KVI2500920	MVs/Texas.USA/12.25/3	D8	9171	2025–03–17
217	TXMEV2500271	KVI2500923	MVs/Texas.USA/Mar.25/1627	D8	9274	2025–03–17
218	TXMEV2500272	KVI2500924	MVs/Texas.USA/Mar.25/1537	D8	9171	2025–03–17
219	TXMEV2500273	KVI2500925	MVs/Texas.USA/11.25/13	D8	9171	2025–03–17
220	TXMEV2500277	KVI2500929	MVs/Texas.USA/12.25/13	D8	not available	2025–03–17
221	TXMEV2500279	KVI2500932	MVs/Texas.USA/Mar.25/1686	D8	9171	2025–03–17
222	TXMEV2500281	KVI2500936	MVs/Texas.USA/Mar.25/1699	D8	9274	2025–03–17
223	TXMEV2500283	KVI2500939	MVs/Texas.USA/Mar.25/1260	D8	9171	2025–03–17
224	TXMEV2500284	KVI2500940	MVs/Texas.USA/Mar.25/1304	D8	9171	2025–03–17
225	TXMEV2500285	KVI2500941	MVs/Texas.USA/12.25/4	D8	not available	2025–03–16
226	TXMEV2500286	KVI2500946	MVs/Texas.USA/Mar.25/1589	D8	9171	2025–03–17
227	TXMEV2500287	KVI2500948	MVs/Texas.USA/12.25/22	D8	not available	2025–03–18
228	TXMEV2500288	KVI2500949	MVs/Texas.USA/12.25/23	D8	not available	2025–03–18
229	TXMEV2500289	KVI2500950	MVs/Texas.USA/12.25/24	D8	not available	2025–03–18
230	TXMEV2500290	KVI2500952	MVs/Texas.USA/Mar.25/1796	D8	9171	2025–03–18
231	TXMEV2500292	KVI2500961	MVs/Texas.USA/Mar.25/1687	D8	9171	2025–03–18
232	TXMEV2500297	KVI2500978	MVs/Texas.USA/12.25/30	D8	not available	2025–03–19
233	TXMEV2500299	KVI2500988	MVs/Texas.USA/Mar.25/1280	D8	9171	2025–03–19
234	TXMEV2500300	KVI2500990	MVs/Texas.USA/Mar.25/1227	D8	9171	2025–03–20
235	TXMEV2500302	KVI2500992	MVs/Texas.USA/Mar.25/1206	D8	9171	2025–03–20
236	TXMEV2500303	KVI2500993	MVs/Texas.USA/Mar.25/1011	D8	9171	2025–03–20
237	TXMEV2500304	KVI2500994	MVs/Texas.USA/12.25/38	D8	not available	2025–03–20
238	TXMEV2500308	KVI2501011	MVs/Texas.USA/Mar.25/1244	D8	9171	2025–03–21
239	TXMEV2500309	KVI2501012	MVs/Texas.USA/Mar.25/1020	D8	9171	2025–03–21
240	TXMEV2500310	KVI2501013	MVs/Texas.USA/Mar.25/1202	D8	9171	2025–03–21
241	TXMEV2500311	KVI2501014	MVs/Texas.USA/Mar.25/1626	D8	9171	2025–03–21
242	TXMEV2500313	KVI2501016	MVs/Texas.USA/Mar.25/1247	D8	9342	2025–03–23
243	TXMEV2500314	KVI2501019	MVs/Texas.USA/Mar.25/1574	D8	9171	2025–03–23
244	TXMEV2500315	KVI2501021	MVs/Texas.USA/Mar.25/1326	D8	9171	2025–03–23
245	TXMEV2500317	KVI2501023	MVs/Texas.USA/Mar.25/1302	D8	9171	2025–03–24

Index	ID	Alternative ID	CDC/WHO Strain name	clade	DSId	date
246	TXMEV2500318	KVI2501024	MVs/Texas.USA/Mar.25/1089	D8	9171	2025–03–23
247	TXMEV2500319	KVI2501028	MVs/Texas.USA/Mar.25/1223	D8	9171	2025–03–23
248	TXMEV2500320	KVI2501030	MVs/Texas.USA/Mar.25/1054	D8	9171	2025–03–24
249	TXMEV2500321	KVI2501032	MVs/Texas.USA/Mar.25/1116	D8	9171	2025–03–23
250	TXMEV2500322	KVI2501033	MVs/Texas.USA/Mar.25/1063	D8	9171	2025–03–24
251	TXMEV2500324	KVI2501035	MVs/Texas.USA/Mar.25/1738	D8	9171	2025–03–24
252	TXMEV2500327	KVI2501073	MVs/Texas.USA/Mar.25/1397	D8	9171	2025–03–25
253	TXMEV2500330	KVI2501076	MVs/Texas.USA/Mar.25/1595	D8	9171	2025–03–25
254	TXMEV2500331	KVI2501078	MVs/Texas.USA/Mar.25/1660	D8	9171	2025–03–25
255	TXMEV2500332	KVI2501079	MVs/Texas.USA/Mar.25/1079	D8	9171	2025–03–25
256	TXMEV2500333	KVI2501080	MVs/Texas.USA/Mar.25/1413	D8	9171	2025–03–24
257	TXMEV2500335	KVI2501086	MVs/Texas.USA/Mar.25/1780	D8	9171	2025–03–26
258	TXMEV2500336	KVI2501088	MVs/Texas.USA/Mar.25/1345	D8	9171	2025–03–26
259	TXMEV2500337	KVI2501089	MVs/Texas.USA/Mar.25/1341	D8	9322	2025–03–26
260	TXMEV2500338	KVI2501098	MVs/Texas.USA/Mar.25/1319	D8	9171	2025–03–21
261	TXMEV2500339	KVI2501109	MVs/Texas.USA/Mar.25/1187	D8	9171	2025–03–27
262	TXMEV2500340	KVI2501116	MVs/Texas.USA/Mar.25/1085	D8	9322	2025–03–27
263	TXMEV2500341	KVI2501117	MVs/Texas.USA/Mar.25/1237	D8	9171	2025–03–27
264	TXMEV2500342	KVI2501118	MVs/Texas.USA/Mar.25/1196	D8	9171	2025–03–27
265	TXMEV2500343	KVI2501119	MVs/Texas.USA/Mar.25/1423	D8	9171	2025–03–26
266	TXMEV2500345	KVI2501122	MVs/Texas.USA/Mar.25/1712	D8	9171	2025–03–30
267	TXMEV2500346	KVI2501124	MVs/Texas.USA/Mar.25/1478	D8	9171	2025–03–28
268	TXMEV2500347	KVI2501125	MVs/Texas.USA/Mar.25/1782	D8	9171	2025–03–28
269	TXMEV2500348	KVI2501127	MVs/Texas.USA/Mar.25/1402	D8	9171	2025–03–30
270	TXMEV2500349	KVI2501130	MVs/Texas.USA/14.25/3	D8	not available	2025–03–30
271	TXMEV2500350	KVI2501131	MVs/Texas.USA/13.25/31	D8	not available	2025–03–28
272	TXMEV2500351	KVI2501132	MVs/Texas.USA/Mar.25/1453	D8	9171	2025–03–28
273	TXMEV2500352	KVI2501133	MVs/Texas.USA/13.25/33	D8	not available	2025–03–28
274	TXMEV2500353	KVI2501134	MVs/Texas.USA/13.25/34	D8	not available	2025–03–28
275	TXMEV2500354	KVI2501135	MVs/Texas.USA/Mar.25/1754	D8	9171	2025–03–30
276	TXMEV2500355	KVI2501144	MVs/Texas.USA/Mar.25/1788	D8	9171	2025–03–30
277	TXMEV2500356	KVI2501145	MVs/Texas.USA/Mar.25/1769	D8	9171	2025–03–30
278	TXMEV2500359	KVI2501150	MVs/Texas.USA/14.25/7	D8	not available	2025–03–30
279	TXMEV2500360	KVI2501153	MVs/Texas.USA/13.25/35	D8	not available	2025–03–28
280	TXMEV2500361	KVI2501154	MVs/Texas.USA/13.25/38	D8	not available	2025–03–29
281	TXMEV2500362	KVI2501155	MVs/Texas.USA/Apr.25/1412	D8	9171	2025–03–31
282	TXMEV2500363	KVI2501157	MVs/Texas.USA/14.25/12	D8	not available	2025–03–31
283	TXMEV2500364	KVI2501159	MVs/Texas.USA/Mar.25/1596	D8	9322	2025–03–28
284	TXMEV2500366	KVI2501161	MVs/Texas.USA/14.25/13	D8	not available	2025–03–31
285	TXMEV2500367	KVI2501163	MVs/Texas.USA/Mar.25/1269	D8	9171	2025–03–27
286	TXMEV2500368	KVI2501164	MVs/Texas.USA/Mar.25/1695	D8	9171	2025–03–28
287	TXMEV2500369	KVI2501165	MVs/Texas.USA/14.25/8	D8	not available	2025–03–30
288	TXMEV2500370	KVI2501169	MVs/Texas.USA/Mar.25/1024	D8	9171	2025–03–30
289	TXMEV2500372	KVI2501183	MVs/Texas.USA/Mar.25/1014	D8	9171	2025–03–30
290	TXMEV2500373	KVI2501187	MVs/Texas.USA/Mar.25/1039	D8	9171	2025–03–31
291	TXMEV2500374	KVI2501188	MVs/Texas.USA/12.25/46	D8	not available	2025–03–22
292	TXMEV2500375	KVI2501189	MVs/Texas.USA/12.25/47	D8	not available	2025–03–22
293	TXMEV2500377	KVI2501191	MVs/Texas.USA/Apr.25/1603	D8	9171	2025–04–01
294	TXMEV2500381	KVI2501200	MVs/Texas.USA/Apr.25/1447	D8	9171	2025–04–01
295	TXMEV2500382	KVI2501202	MVs/Texas.USA/Mar.25/1808	D8	9171	2025–03–31
296	TXMEV2500383	KVI2501213	MVs/Texas.USA/14.25/20	D8	not available	2025–04–02
297	TXMEV2500384	KVI2501215	MVs/Texas.USA/Apr.25/1792	D8	9171	2025–04–02
298	TXMEV2500385	KVI2501220	MVs/Texas.USA/Apr.25/1208	D8	9171	2025–04–02
299	TXMEV2500386	KVI2501221	MVs/Texas.USA/14.25/23	D8	not available	2025–04–02
300	TXMEV2500390	KVI2501229	MVs/Texas.USA/14.25/24	D8	not available	2025–04–03
301	TXMEV2500391	KVI2501234	MVs/Texas.USA/Apr.25/1228	D8	9171	2025–04–03
302	TXMEV2500392	KVI2501238	MVs/Texas.USA/Apr.25/1519	D8	9171	2025–04–04
303	TXMEV2500393	KVI2501239	MVs/Texas.USA/Apr.25/1711	D8	9171	2025–04–04
304	TXMEV2500394	KVI2501245	MVs/Texas.USA/Apr.25/1282	D8	9171	2025–04–04
305	TXMEV2500395	KVI2501249	MVs/Texas.USA/Apr.25/1391	D8	9171	2025–04–06
306	TXMEV2500396	KVI2501262	MVs/Texas.USA/Apr.25/1475	D8	9171	2025–04–07
307	TXMEV2500398	KVI2501264	MVs/Texas.USA/15.25/6	D8	not available	2025–04–07
308	TXMEV2500399	KVI2501265	MVs/Texas.USA/15.25/7	D8	not available	2025–04–07
309	TXMEV2500402	KVI2501294	MVs/Texas.USA/Apr.25/1619	D8	9171	2025–04–08
310	TXMEV2500404	KVI2501297	MVs/Texas.USA/Apr.25/1577	D8	9171	2025–04–08
311	TXMEV2500405	KVI2501299	MVs/Texas.USA/Apr.25/1139	D8	9171	2025–04–08
312	TXMEV2500408	KVI2501327	MVs/Texas.USA/Apr.25/1441	D8	9171	2025–04–07
313	TXMEV2500411	KVI2501349	MVs/Texas.USA/Apr.25/1115	D8	9377	2025–04–11
314	TXMEV2500412	KVI2501357	MVs/Texas.USA/Apr.25/1153	D8	9171	2025–04–11

Index	ID	Alternative ID	CDC/WHO Strain name	clade	DSId	date
315	TXMEV2500413	KVI2501360	MVs/Texas.USA/Apr.25/1236	D8	9274	2025–04–12
316	TXMEV2500414	KVI2501373	MVs/Texas.USA/Apr.25/1229	D8	9171	2025–04–11
317	TXMEV2500416	KVI2501377	MVs/Texas.USA/Apr.25/1233	D8	9171	2025–04–11
318	TXMEV2500417	KVI2501379	MVs/Texas.USA/Apr.25/1147	D8	9421	2025–04–13
319	TXMEV2500420	KVI2501396	MVs/Texas.USA/Apr.25/1710	D8	9171	2025–04–14
320	TXMEV2500421	KVI2501397	MVs/Texas.USA/Apr.25/1805	D8	9171	2025–04–10
321	TXMEV2500424	KVI2501415	MVs/Texas.USA/Apr.25/1690	D8	9171	2025–04–15
322	TXMEV2500425	KVI2501434	MVs/Texas.USA/Apr.25/1254	D8	9171	2025–04–16
323	TXMEV2500426	KVI2501439	MVs/Texas.USA/Apr.25/1318	D8	9171	2025–04–16
324	TXMEV2500427	KVI2501440	MVs/Texas.USA/Apr.25/1400	D8	9171	2025–04–16
325	TXMEV2500428	KVI2501444	MVs/Texas.USA/Apr.25/1461	D8	9171	2025–04–17
326	TXMEV2500429	KVI2501455	MVs/Texas.USA/16.25/12	D8	not available	2025–04–17
327	TXMEV2500431	KVI2501462	MVs/Texas.USA/Apr.25/1694	D8	9171	2025–04–17
328	TXMEV2500432	KVI2501464	MVs/Texas.USA/Apr.25/1415	D8	9171	2025–04–16
329	TXMEV2500434	KVI2501475	MVs/Texas.USA/16.25/23	D8	not available	2025–04–19
330	TXMEV2500435	KVI2501477	MVs/Texas.USA/Apr.25/1527	D8	9450	2025–04–18
331	TXMEV2500436	KVI2501482	MVs/Texas.USA/Apr.25/1097	D8	9171	2025–04–18
332	TXMEV2500437	KVI2501485	MVs/Texas.USA/16.25/16	D8	not available	2025–04–18
333	TXMEV2500438	KVI2501487	MVs/Texas.USA/Apr.25/1497	D8	9377	2025–04–20
334	TXMEV2500439	KVI2501488	MVs/Texas.USA/16.25/24	D8	not available	2025–04–19
335	TXMEV2500440	KVI2501491	MVs/Texas.USA/16.25/17	D8	not available	2025–04–18
336	TXMEV2500441	KVI2501492	MVs/Texas.USA/16.25/18	D8	not available	2025–04–18
337	TXMEV2500442	KVI2501493	MVs/Texas.USA/16.25/19	D8	not available	2025–04–18
338	TXMEV2500445	KVI2501502	MVs/Texas.USA/Apr.25/1590	D8	9171	2025–04–21
339	TXMEV2500446	KVI2501512	MVs/Texas.USA/17.25/4	D8	not available	2025–04–22
340	TXMEV2500447	KVI2501515	MVs/Texas.USA/Apr.25/1110	D8	9171	2025–04–22
341	TXMEV2500449	KVI2501538	MVs/Texas.USA/Apr.25/1615	D8	9171	2025–04–24
342	TXMEV2500450	KVI2501544	MVs/Texas.USA/Apr.25/1759	D8	9171	2025–04–24
343	TXMEV2500451	KVI2501574	MVs/Texas.USA/Apr.25/1514	D8	9171	2025–04–25
344	TXMEV2500454	KVI2501595	MVs/Texas.USA/Apr.25/1195	D8	9171	2025–04–29
345	TXMEV2500455	KVI2501600	MVs/Texas.USA/Apr.25/1211	D8	9171	2025–04–29
346	TXMEV2500457	KVI2501605	MVs/Texas.USA/Apr.25/1050	D8	9171	2025–04–30
347	TXMEV2500458	KVI2501608	MVs/Texas.USA/Apr.25/1798	D8	9171	2025–04–30
348	TXMEV2500462	KVI2501647	MVs/Texas.USA/May.25/1264	D8	9171	2025–05–01
349	TXMEV2500464	KVI2501651	MVs/Texas.USA/May.25/1138	D8	9171	2025–05–06
350	TXMEV2500465	KVI2501652	MVs/Texas.USA/19.25/3	D8	not available	2025–05–06
351	TXMEV2500467	KVI2501662	MVs/Texas.USA/19.25/5	D8	not available	2025–05–07
352	TXMEV2500468	KVI2501667	MVs/Texas.USA/May.25/1562	D8	9171	2025–05–07
353	TXMEV2500469	KVI2501669	MVs/Texas.USA/May.25/1385	D8	9171	2025–05–08
354	TXMEV2500471	AVI2501744	MVs/Texas.USA/May.25/1134	D8	9171	2025–05–16
355	TXMEV2500472	AVI2501746	MVs/Texas.USA/May.25/1579	D8	9171	2025–05–18
356	TXMEV2500474	AVI2501750	MVs/Texas.USA/May.25/1361	D8	9329	2025–05–19
357	TXMEV2500476	AVI2501774	MVs/Texas.USA/May.25/1731	D8	9171	2025–05–27
358	TXMEV2500477	AVI2501783	MVs/Texas.USA/Jun.25/1756	D8	9171	2025–06–02
359	TXMEV2500478	AVI2501806	MVs/Texas.USA/Jun.25/1548	D8	9171	2025–06–16
360	TXMEV2500480	294893	MVs/Texas.USA/May.25/1662	D8	9171	2025–05–02
361	TXMEV2500482	KVI2501765	MVs/Texas.USA/May.25/1678	D8	9171	2025–05–26
362	TXMEV2500483	KVI2501769	MVs/Texas.USA/May.25/1592	D8	9171	2025–05–26
363	TXMEV2500484	KVI2501784	MVs/Texas.USA/Jun.25/1797	D8	9171	2025–06–04
364	TXMEV2500485	AVI2501818	MVs/Texas.USA/Jun.25/1133	D8	9171	2025–06–23
365	TXMEV2500488	AVI2501822	MVs/Texas.USA/Jul.25/1033	D8	9171	2025–07–01
366	TXMEV2500489	AVI2501832	MVs/Texas.USA/Jul.25/1701	D8	9171	2025–07–23
367	TXMEV2500491	KVI2501845	MVs/Texas.USA/Jul.25/1643	D8	9461	2025–07–30
368	TXMEV2500492	AVI2501846	MVs/Texas.USA/Aug.25/1709	D8	10224	2025–08–19

Appendix Table 2. Demographic and epidemiologic data for the 368 sequences, represented by 359 individuals.

Characteristic	Count (n = 359)	Percent (%)
Age Group		
0–6 mo	14	3.90
7–11 mo	22	6.13
1–4 y	83	23.12
5–17 y	127	35.38
18+ years	113	31.48
Sex		
Female	173	48.19
Male	186	51.81
Pregnancy among Females (n = 173)		
Yes	7	4.05
No	2	1.16
Unknown	164	94.80
Vaccine History		
Vaccinated: 1 dose	7	1.95
Vaccinated: 2 doses	9	2.51
No	204	56.82
Unknown	139	38.72
Exposure Setting		
Church	3	0.84
Daycare/Childcare Center	7	1.95
Home	71	19.78
Hospital/ER	1	0.28
Other	31	8.64
School	42	11.70
Work	10	2.79
Unknown	194	54.04
Travel before Onset		
Yes	56	15.60
No	138	38.44
Unknown	165	45.96
ER Only		
Yes	61	16.99
No	23	6.41
Unknown	275	76.60
Hospitalized		
Yes	72	20.06
No	116	32.31
Unknown	171	47.63
ICU Admission		
Yes	8	2.23
No	123	34.26
Unknown	228	63.51
Fever		
Yes	317	88.30
No	10	2.79
Unknown	32	8.91
Rash		
Yes	320	89.14
No	12	3.34
Unknown	27	7.52

Appendix Table 3. Publicly available genotype D8 whole genomes screened for F:H419R (2023–01–01 through 2025–09–24).

SeqName	Clade	Date	mutation
PX308137.1	MVs/Utah.USA/31.25/6[D8]	2025–08–01	F:H419R
PX308138.1	MVs/Utah.USA/31.25/2[D8]	2025–08–01	F:H419R
PX308139.1	MVs/Utah.USA/31.25/5[D8]	2025–08–01	F:H419R
PX308140.1	MVs/Utah.USA/31.25/4[D8]	2025–08–01	F:H419R
PX308141.1	MVs/Utah.USA/31.25/3[D8]	2025–08–01	F:H419R
PX308142.1	MVs/Utah.USA/31.25[D8]	2025–08–01	F:H419R
PX308143.1	MVs/Utah.USA/31.25/7[D8]	2025–08–01	F:H419R
PX308144.1	MVs/Utah.USA/31.25/9[D8]	2025–08–01	F:H419R
PX308145.1	MVs/Utah.USA/31.25/13[D8]	2025–08–01	F:H419R
PX308146.1	MVs/Utah.USA/31.25/8[D8]	2025–08–01	F:H419R
PX308147.1	MVs/Utah.USA/31.25/10[D8]	2025–08–01	F:H419R
PX308148.1	MVs/Utah.USA/31.25/11[D8]	2025–08–01	F:H419R
PX308149.1	MVs/Utah.USA/31.25/14[D8]	2025–08–01	F:H419R
PX308150.1	MVs/Utah.USA/31.25/12[D8]	2025–08–01	F:H419R
PX121936.1	MVs/Utah.USA/27.25[D8]	2025–07–01	F:H419R
PV870363.1	MVs/Zuid-Holland.NLD/24.25[D8]	2025–06–17	F:H419R
PV870360.1	MVs/Utrecht.NLD/24.25[D8]	2025–06–11	
PV870361.1	MVs/Utrecht.NLD/24.25/2[D8]	2025–06–11	
PV870362.1	MVs/Zuid-Holland.NLD/23.25/3[D8]	2025–06–06	
PV870359.1	MVs/Utrecht.NLD/23.25[D8]	2025–06–05	
PV809720.1	MVs/Zuid-Holland.NLD/23.25[D8]	2025–06–03	F:H419R
PV809721.1	MVs/Gelderland.NLD/23.25[D8]	2025–06–03	F:H419R
PX121931.1	MVs/Utah.USA/23.25/2[D8]	2025–06–01	F:H419R
PX121932.1	MVs/Utah.USA/23.25/4[D8]	2025–06–01	
PX121933.1	MVs/Utah.USA/23.25/3[D8]	2025–06–01	
PX121934.1	MVs/Utah.USA/23.25[D8]	2025–06–01	F:H419R
PX121935.1	MVs/Utah.USA/23.25/5[D8]	2025–06–01	F:H419R
PV870357.1	MVs/Utrecht.NLD/21.25[D8]	2025–05–26	
PV870365.1	MVs/Utrecht.NLD/21.25/2[D8]	2025–05–26	
PV870366.1	MVs/Zuid-Holland.NLD/21.25/2[D8]	2025–05–24	
PV870358.1	MVs/Zeeland.NLD/20.25[D8]	2025–05–18	
PV870356.1	MVs/Gelderland.NLD/20.25[D8]	2025–05–16	
PV686489.1	MVs/California.USA/17.25[D8]	2025–04–23	F:H419R
PV870351.1	MVs/Noord-Holland.NLD/14.25[D8]	2025–04–07	F:H419R
PV870350.1	MVs/Noord-Brabant.NLD/12.25[D8]	2025–03–17	F:H419R
PV870349.1	MVs/Noord-Brabant.NLD/11.25/2[D8]	2025–03–14	F:H419R
PV870354.1	MVs/Noord-Brabant.NLD/11.25/3[D8]	2025–03–14	F:H419R
PV870348.1	MVs/Noord-Brabant.NLD/10.25[D8]	2025–03–05	F:H419R
PV870347.1	MVs/Noord-Brabant.NLD/9.25/3[D8]	2025–03–03	F:H419R
PV870344.1	MVs/Noord-Brabant.NLD/9.25/2[D8]	2025–02–26	
PV870338.1	MVs/Noord-Brabant.NLD/8.25[D8]	2025–02–25	F:H419R
PV870345.1	MVs/Noord-Brabant.NLD/8.25/4[D8]	2025–02–25	F:H419R
PV870346.1	MVs/Noord-Brabant.NLD/9.25[D8]	2025–02–25	F:H419R
PV870343.1	MVs/Noord-Brabant.NLD/8.25/3[D8]	2025–02–24	F:H419R
PV870342.1	MVs/Noord-Brabant.NLD/8.25/2[D8]	2025–02–19	F:H419R
PV870353.1	MVs/Noord-Brabant.NLD/7.25[D8]	2025–02–19	F:H419R
PV870340.1	MVs/Friesland.NLD/5.25[D8]	2025–02–13	
PV870341.1	MVs/Friesland.NLD/4.25[D8]	2025–01–22	
PV870339.1	MVs/Noord-Brabant.NLD/3.25[D8]	2025–01–16	
PV870352.1	MVs/Noord-Brabant.NLD/1.25/2[D8]	2025–01–04	
PQ590060.1	MVs/SaoPaulo.BRA/41.24[D8]	2024–10–11	F:H419R
PQ786194.1	MVs/Neunkirchen.AUT/13.24[D8]	2024–03–23	
PQ786225.1	MVs/Wien.AUT/12.24/8[D8]	2024–03–20	
PQ786272.1	MVs/Innsbruck.AUT/13.24/2[D8]	2024–03–19	
PQ786269.1	MVs/Innsbruck.AUT/13.24[D8]	2024–03–17	
PQ786178.1	MVs/Wien.AUT/12.24/3[D8]	2024–03–13	
PQ786177.1	MVs/Wien.AUT/12.24[D8]	2024–03–11	
PQ786268.1	MVs/Innsbruck.AUT/12.24/4[D8]	2024–03–11	
PQ786266.1	MVs/Innsbruck.AUT/12.24/2[D8]	2024–03–10	
PQ786267.1	MVs/Innsbruck.AUT/12.24/3[D8]	2024–03–10	
PQ786176.1	MVs/Wien.AUT/11.24/2[D8]	2024–03–09	
PQ786271.1	MVs/Innsbruck.AUT/12.24/5[D8]	2024–03–08	
PQ786175.1	MVs/Wien.AUT/10.24/3[D8]	2024–03–07	
PQ786265.1	MVs/Innsbruck.AUT/11.24[D8]	2024–03–07	
PQ786270.1	MVs/Innsbruck.AUT/12.24[D8]	2024–03–06	
PQ394296.1	MVs/Rome.ITA/10.24/12[D8]	2024–03–04	
PQ786212.1	MVs/Innsbruck.AUT/11.24/8[D8]	2024–03–04	
PQ394295.1	MVs/Rome.ITA/9.24/11[D8]	2024–03–03	

SeqName	Clade	Date	mutation
PQ786211.1	MVs/Innsbruck.AUT/11.24/2[D8]	2024–03–03	
PQ786210.1	MVs/Innsbruck.AUT/11.24/6[D8]	2024–03–02	
PQ786264.1	MVs/Innsbruck.AUT/11.24/9[D8]	2024–03–02	
PQ786174.1	MVs/Wien.AUT/09.24/11[D8]	2024–03–01	
PQ394293.1	MVs/Rome.ITA/9.24/09[D8]	2024–02–29	
PQ394294.1	MVs/Rome.ITA/9.24/10[D8]	2024–02–29	
PQ786209.1	MVs/Innsbruck.AUT/11.24/4[D8]	2024–02–29	
PQ786262.1	MVs/Innsbruck.AUT/11.24/3[D8]	2024–02–29	
PQ786263.1	MVs/Innsbruck.AUT/11.24/5[D8]	2024–02–29	
PQ394292.1	MVs/Rome.ITA/9.24/08[D8]	2024–02–28	
PQ786171.1	MVs/Wien.AUT/09.24[D8]	2024–02–28	
PQ786172.1	MVs/Wien.AUT/09.24/2[D8]	2024–02–28	
PQ786180.1	MVs/Feldkirch.AUT/10.24/5[D8]	2024–02–27	
PQ786170.1	MVs/Wien.AUT/08.24/7[D8]	2024–02–26	
PQ786173.1	MVs/Wien.AUT/09.24/7[D8]	2024–02–26	
PQ786260.1	MVs/Innsbruck.AUT/10.24/3[D8]	2024–02–26	
PQ786261.1	MVs/Innsbruck.AUT/10.24/4[D8]	2024–02–26	
PQ786259.1	MVs/Innsbruck.AUT/10.24/2[D8]	2024–02–25	
PQ394291.1	MVs/Rome.ITA/8.24/07[D8]	2024–02–24	
PQ786168.1	MVs/Wien.AUT/08.24/2[D8]	2024–02–24	
PQ786258.1	MVs/Innsbruck.AUT/10.24[D8]	2024–02–24	
PQ786257.1	MVs/Innsbruck.AUT/10.24/5[D8]	2024–02–23	
PQ394290.1	MVs/Rome.ITA/8.24/06[D8]	2024–02–22	
PQ786169.1	MVs/Wien.AUT/08.24/3[D8]	2024–02–19	
PQ786208.1	MVs/Innsbruck.AUT/07.24/11[D8]	2024–02–18	
PQ786253.1	MVs/Innsbruck.AUT/07.24/19[D8]	2024–02–18	
PQ786254.1	MVs/Innsbruck.AUT/07.24/14[D8]	2024–02–18	
PQ786255.1	MVs/Innsbruck.AUT/07.24/13[D8]	2024–02–18	
PQ786256.1	MVs/Innsbruck.AUT/07.24/17[D8]	2024–02–18	
PQ786167.1	MVs/Wien.AUT/07.24/4[D8]	2024–02–17	
PQ786252.1	MVs/Innsbruck.AUT/07.24/15[D8]	2024–02–17	
PQ786251.1	MVs/Innsbruck.AUT/07.24/18[D8]	2024–02–16	
PP626047.1	MVs/Pattani.THA/07.2024[D8]	2024–02–16	F:H419R
PQ786250.1	MVs/Innsbruck.AUT/07.24/4[D8]	2024–02–15	
PQ786207.1	MVs/Kufstein.AUT/07.24[D8]	2024–02–14	
PQ786248.1	MVs/Innsbruck.AUT/07.24/5[D8]	2024–02–14	
PQ786249.1	MVs/Innsbruck.AUT/08.24/2[D8]	2024–02–14	
PQ786273.1	MVs/Innsbruck.AUT/07.24/10[D8]	2024–02–14	
PQ394288.1	MVs/Rome.ITA/7.24/05[D8]	2024–02–13	F:H419R
PQ786206.1	MVs/Innsbruck.AUT/07.24/3[D8]	2024–02–13	
PQ786245.1	MVs/Innsbruck.AUT/07.24[D8]	2024–02–13	
PQ786246.1	MVs/Innsbruck.AUT/07.24/7[D8]	2024–02–13	
PQ786247.1	MVs/Innsbruck.AUT/07.24/9[D8]	2024–02–13	
PQ394287.1	MVs/Rome.ITA/7.24/04[D8]	2024–02–12	
PQ786205.1	MVs/Innsbruck.AUT/07.24/2[D8]	2024–02–12	
PQ786244.1	MVs/Innsbruck.AUT/07.24/6[D8]	2024–02–12	
PQ394289.1	MVs/Rome.ITA/6.24/03[D8]	2024–02–11	
PQ786204.1	MVs/Innsbruck.AUT/06.24/7[D8]	2024–02–11	
PQ786239.1	MVs/Koessen.AUT/07.24[D8]	2024–02–11	
PQ786240.1	MVs/Innsbruck.AUT/06.24/8[D8]	2024–02–11	
PQ786241.1	MVs/Innsbruck.AUT/06.24/9[D8]	2024–02–11	
PQ786242.1	MVs/Innsbruck.AUT/06.24/10[D8]	2024–02–11	
PQ786243.1	MVs/Innsbruck.AUT/06.24/11[D8]	2024–02–11	
PQ786166.1	MVs/Wien.AUT/06.24/9[D8]	2024–02–10	
PQ786238.1	MVs/Innsbruck.AUT/07.24/8[D8]	2024–02–10	
PQ786165.1	MVs/Wien.AUT/06.24/8[D8]	2024–02–08	
PQ786162.1	MVs/Wien.AUT/06.24/4[D8]	2024–02–07	
PQ786163.1	MVs/Wien.AUT/06.24/5[D8]	2024–02–07	
PQ786164.1	MVs/Wien.AUT/06.24/7[D8]	2024–02–06	
PQ786202.1	MVs/Innsbruck.AUT/06.24/2[D8]	2024–02–06	
PQ786203.1	MVs/Innsbruck.AUT/06.24/3[D8]	2024–02–06	
PQ786201.1	MVs/Innsbruck.AUT/06.24/5[D8]	2024–02–05	
PQ786236.1	MVs/Innsbruck.AUT/06.24[D8]	2024–02–05	
PQ786237.1	MVs/Innsbruck.AUT/06.24/6[D8]	2024–02–05	
PQ786200.1	MVs/Innsbruck.AUT/05.24/10[D8]	2024–02–04	
PQ786231.1	MVs/Innsbruck.AUT/05.24/8[D8]	2024–02–04	
PQ786232.1	MVs/Innsbruck.AUT/05.24/4[D8]	2024–02–04	
PQ786233.1	MVs/Innsbruck.AUT/05.24/9[D8]	2024–02–04	
PQ786234.1	MVs/Innsbruck.AUT/05.24/3[D8]	2024–02–04	

SeqName	Clade	Date	mutation
PQ786235.1	MVs/Innsbruck.AUT/05.24/12[D8]	2024–02–04	
PQ786229.1	MVs/Innsbruck.AUT/05.24/6[D8]	2024–02–03	
PQ786230.1	MVs/Innsbruck.AUT/05.24/7[D8]	2024–02–03	
PQ786160.1	MVs/Wien.AUT/05.24/4[D8]	2024–02–02	
PQ786199.1	MVs/Innsbruck.AUT/05.24/5[D8]	2024–02–02	
PQ786228.1	MVs/Innsbruck.AUT/05.24/2[D8]	2024–02–02	
PQ786227.1	MVs/Innsbruck.AUT/05.24/11[D8]	2024–02–01	
PQ786226.1	MVs/Innsbruck.AUT/06.24/4[D8]	2024–01–31	
PQ786198.1	MVs/Innsbruck.AUT/05.24[D8]	2024–01–30	
PQ786158.1	MVs/Wien.AUT/05.24/2[D8]	2024–01–29	
PQ786156.1	MVs/Wien.AUT/04.24[D8]	2024–01–26	
PQ786159.1	MVs/Wien.AUT/04.24/10[D8]	2024–01–26	
PQ786197.1	MVs/Innsbruck.AUT/04.24/2[D8]	2024–01–26	
PQ786161.1	MVs/Wien.AUT/04.24/12[D8]	2024–01–24	
PQ786196.1	MVs/Innsbruck.AUT/03.24[D8]	2024–01–24	
PQ786195.1	MVs/Innsbruck.AUT/04.24[D8]	2024–01–23	
PQ786157.1	MVs/Wien.AUT/04.24/2[D8]	2024–01–22	
PV872554.1	Mvs/Rome.ITA/3.24/14[D8]	2024–01–18	
PV872555.1	Mvs/Rome.ITA/3.24/13[D8]	2024–01–16	
PQ786155.1	MVs/Wien.AUT/02.24/2[D8]	2024–01–13	
LC848726.1	MVs/KobeC.JPN/31.24/157[D8]	2024–01–01	F:H419R
LC848727.1	MVi/KobeC.JPN/31.24/157[D8]	2024–01–01	F:H419R
LC848728.1	MVs/KobeC.JPN/32.24/170[D8]	2024–01–01	F:H419R
LC848729.1	MVi/KobeC.JPN/32.24/170[D8]	2024–01–01	F:H419R
LC848730.1	MVs/KobeC.JPN/32.24/174[D8]	2024–01–01	F:H419R
LC848731.1	MVi/KobeC.JPN/32.24/174[D8]	2024–01–01	F:H419R
PP868388.1	MVs/Queensland.AUS/04.24[D8]	2024–01–01	F:H419R
PQ453569.1	MVs/LonsLeSaunier.FRA/52.23[D8]	2023–12–29	
PQ786154.1	MVs/Wien.AUT/51.23[D8]	2023–12–20	
PQ394286.1	MVs/Rome.ITA/51.23/02[D8]	2023–12–18	
PQ786153.1	MVs/Wien.AUT/50.23/5[D8]	2023–12–14	
PQ786222.1	MVs/Wien.AUT/50.23[D8]	2023–12–09	
PQ786223.1	MVs/Wien.AUT/50.23/2[D8]	2023–12–09	
PQ786224.1	MVs/Wien.AUT/50.23/3[D8]	2023–12–09	
PQ786152.1	MVs/Wien.AUT/49.23/3[D8]	2023–12–08	
PQ786214.1	MVs/Wien.AUT/49.23/2[D8]	2023–12–07	
PQ394285.1	MVs/Rome.ITA/49.23/01[D8]	2023–12–06	
PQ786220.1	MVs/Wien.AUT/48.23[D8]	2023–11–27	
PQ786221.1	MVs/Wien.AUT/48.23/2[D8]	2023–11–27	
PQ786219.1	MVs/Wien.AUT/47.23[D8]	2023–11–23	
PQ786151.1	MVs/Wien.AUT/46.23/2[D8]	2023–11–18	
PQ786150.1	MVs/Wien.AUT/46.23[D8]	2023–11–16	
PQ786149.1	MVs/Wien.AUT/45.23/3[D8]	2023–11–11	
PQ786147.1	MVs/Wien.AUT/45.23[D8]	2023–11–10	
PQ786148.1	MVs/Wien.AUT/45.23/2[D8]	2023–11–08	
PQ786218.1	MVs/Wien.AUT/45.23/4[D8]	2023–11–08	
PQ786179.1	MVs/Dorbirn.AUT/46.23[D8]	2023–11–07	
PQ786216.1	MVs/Wien.AUT/35.23[D8]	2023–08–30	
PQ786217.1	MVs/Wien.AUT/35.23/2[D8]	2023–08–30	
PQ786215.1	MVs/Wien.AUT/34.23[D8]	2023–08–21	
PP887450.1	MVs/Szeged.HUN/27.23[D8]	2023–07–10	
LC789941.1	MVs/Tokyo.JPN/24.23/23124/[D8]	2023–06–01	F:H419R
OR290099.1	MVs/Moscow.RUS/21.23/1[D8]	2023–05–25	
OR290100.1	MVs/Moscow.RUS/21.23/2[D8]	2023–05–25	
OR290101.1	MVs/Moscow.RUS/20.23/3[D8]	2023–05–25	
OR290102.1	MVs/Moscow.RUS/21.23/3[D8]	2023–05–25	
OR290097.1	MVs/Moscow.RUS/20.23/1[D8]	2023–05–22	
OR854811.1	MVs/Moscow.RUS/19.23[D8]	2023–05–12	
PQ786193.1	MVs/Villach.AUT/18.23[D8]	2023–05–05	
PQ786192.1	MVs/Klagenfurt.AUT/16.23[D8]	2023–04–17	
PQ786188.1	MVs/Graz.AUT/14.23[D8]	2023–04–06	
PQ786191.1	MVs/Graz.AUT/14.23/5[D8]	2023–04–06	
PQ786190.1	MVs/Graz.AUT/14.23/4[D8]	2023–04–04	
PQ786189.1	MVs/Graz.AUT/14.23/3[D8]	2023–04–03	
PQ786187.1	MVs/Graz.AUT/13.23[D8]	2023–03–30	
PQ786186.1	MVs/Graz.AUT/12.23[D8]	2023–03–12	
PQ786185.1	MVs/Graz.AUT/11.23/3[D8]	2023–03–08	
PQ786184.1	MVs/Graz.AUT/9.23/12[D8]	2023–02–27	
PQ786183.1	MVs/Graz.AUT/8.23/2[D8]	2023–02–16	

SeqName	Clade	Date	mutation
PQ786213.1	MVs/Wien.AUT/7.23[D8]	2023–02–16	
OR854812.1	MMVs/Novosibirsk.RUS/07.23[D8]	2023–02–16	
PQ786182.1	MVs/Graz.AUT/8.23[D8]	2023–02–15	
OR805841.2	MVs/Mumbai.IND/05.23/233867[D8]PNEUMONIA	2023–01–30	
PQ786181.1	MVs/Graz.AUT/5.23[D8]	2023–01–24	

Appendix Table 4. Clockor2 root-to-tip regression diagnostics for genotype D8 measles virus datasets. The North American contextual dataset included all 1,771 genomes used for broad phylogenetic placement. The focal expanding North American lineage included 1,760 genomes after excluding 11 contextual genomes outside the focal lineage. Root-to-tip genetic distance was regressed against sample collection date under Clockor2 strict-clock regression. Intercept values from datasets with weak temporal signals were not interpreted as biologically meaningful root dates. These diagnostics are distinct from the TreeTime clock-fit diagnostics shown in Appendix Figure 6.

Clock	#Tips	Rate	R^2	Residual mean squared	Intercept
North American contextual dataset	1771	7.389×10^{-4}	0.465	8.502×10^{-8}	2007.227
Focal expanding North American lineage	1760	7.189×10^{-4}	0.495	6.924×10^{-8}	2006.702
Utah	371	3.995×10^{-4}	0.059	8.415×10^{-8}	1990.729
South Carolina	369	1.551×10^{-4}	0.032	4.260×10^{-9}	1938.099
Texas	368	1.723×10^{-4}	0.003	6.179×10^{-8}	1948.036
Arizona	104	3.288×10^{-4}	0.06	4.444×10^{-8}	1983.065

Appendix Table 5. Pairwise SNP distances and callable-site overlap within and between North American genotype D8 datasets.

Comparison group	Number of Pairs	Min SNP	Q1 SNP	Median SNP	Mean SNP	Q3 SNP	Max SNP	median callable sites	median callable reference %
Texas vs Texas	65184	0	2	3	3.49	5	15	14020	88.21
Utah vs Utah	61347	0	6	12	12.38	18	36	15022	94.51
Arizona vs Arizona	4817	0	3	5	6.50	8	28	14946	94.04
South Carolina vs South Carolina	62814	0	1	1	1.69	2	15	14387	90.52
Texas vs Utah	125976	1	11	15	15.12	19	34	14252	89.67
Texas vs Arizona	35214	2	14	16	15.17	17	29	14263	89.74
Texas vs South Carolina	126054	3	8	9	9.35	10	20	14023	88.23

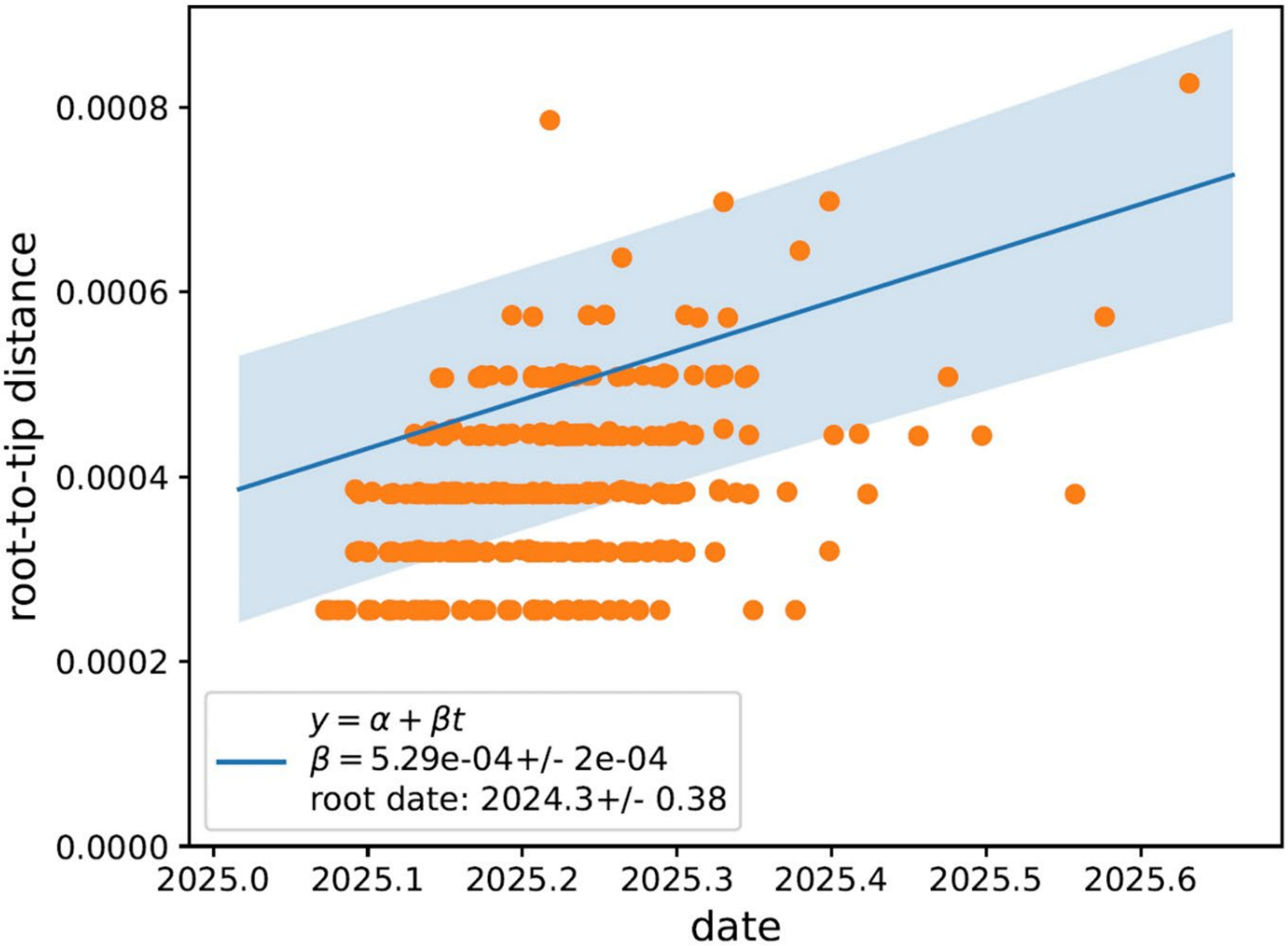

Appendix Figure 1. Root-to-tip regression used for molecular-clock assessment of the 2025 Texas measles genomes. Root-to-tip genetic distance was plotted against sampling date for the 2025 Texas measles genome dataset (n = 368). The fitted regression estimated a clock rate of 5.29×10^{-4} substitutions/site/year. The regression R^2 was 0.15.

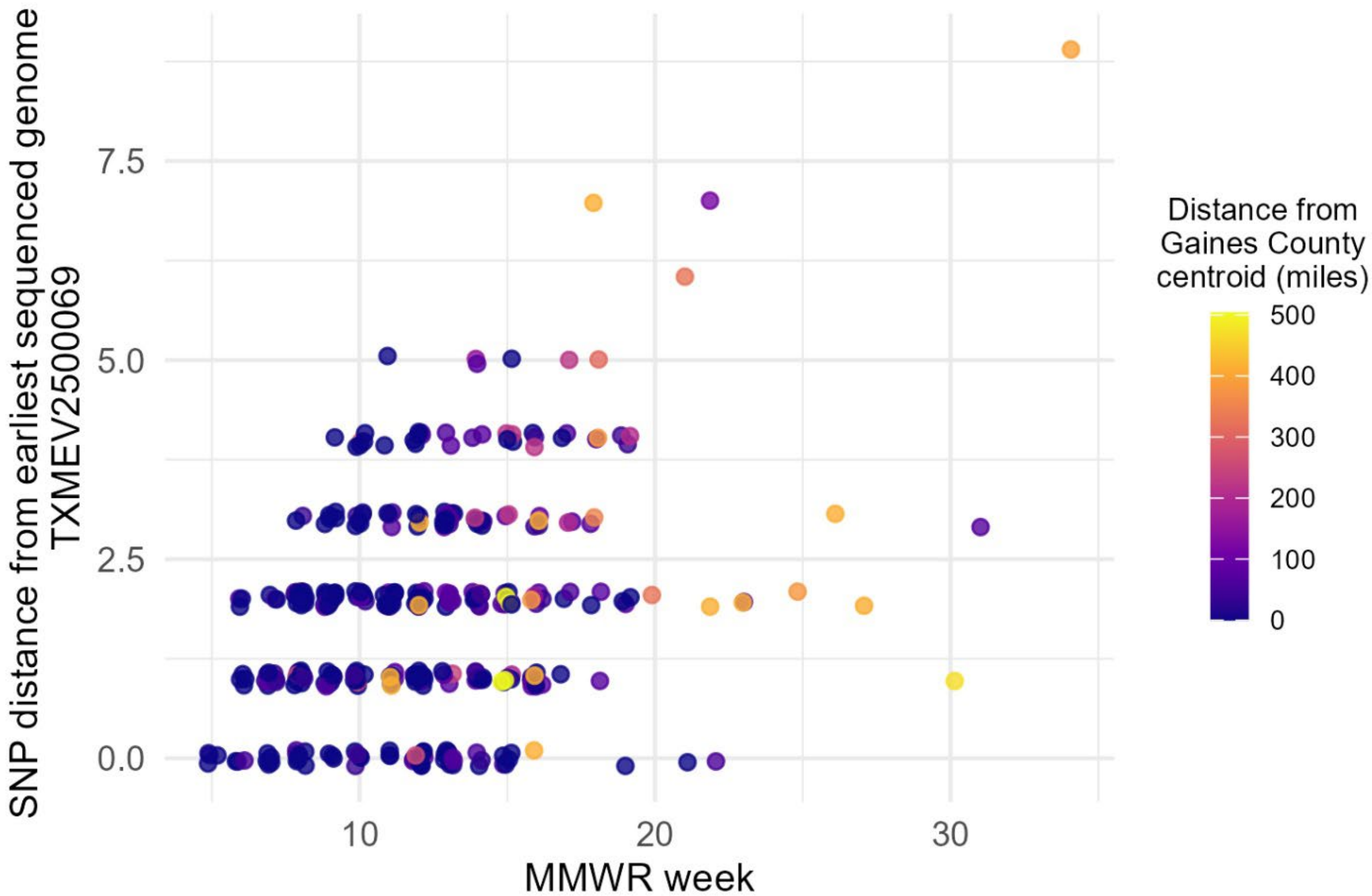

Appendix Figure 2. Genetic diversification and geographic spread of measles virus genomes during the Texas outbreak. Each point represents a sequenced genome. The x-axis shows epidemiologic week of specimen collection (MMWR week), and the y-axis indicates single-nucleotide polymorphism (SNP) distance from TXMEV2500069, the earliest sequenced genome in the Texas dataset. Point color represents the geographic distance, in miles, between the county of specimen collection and the centroid of Gaines County, Texas.

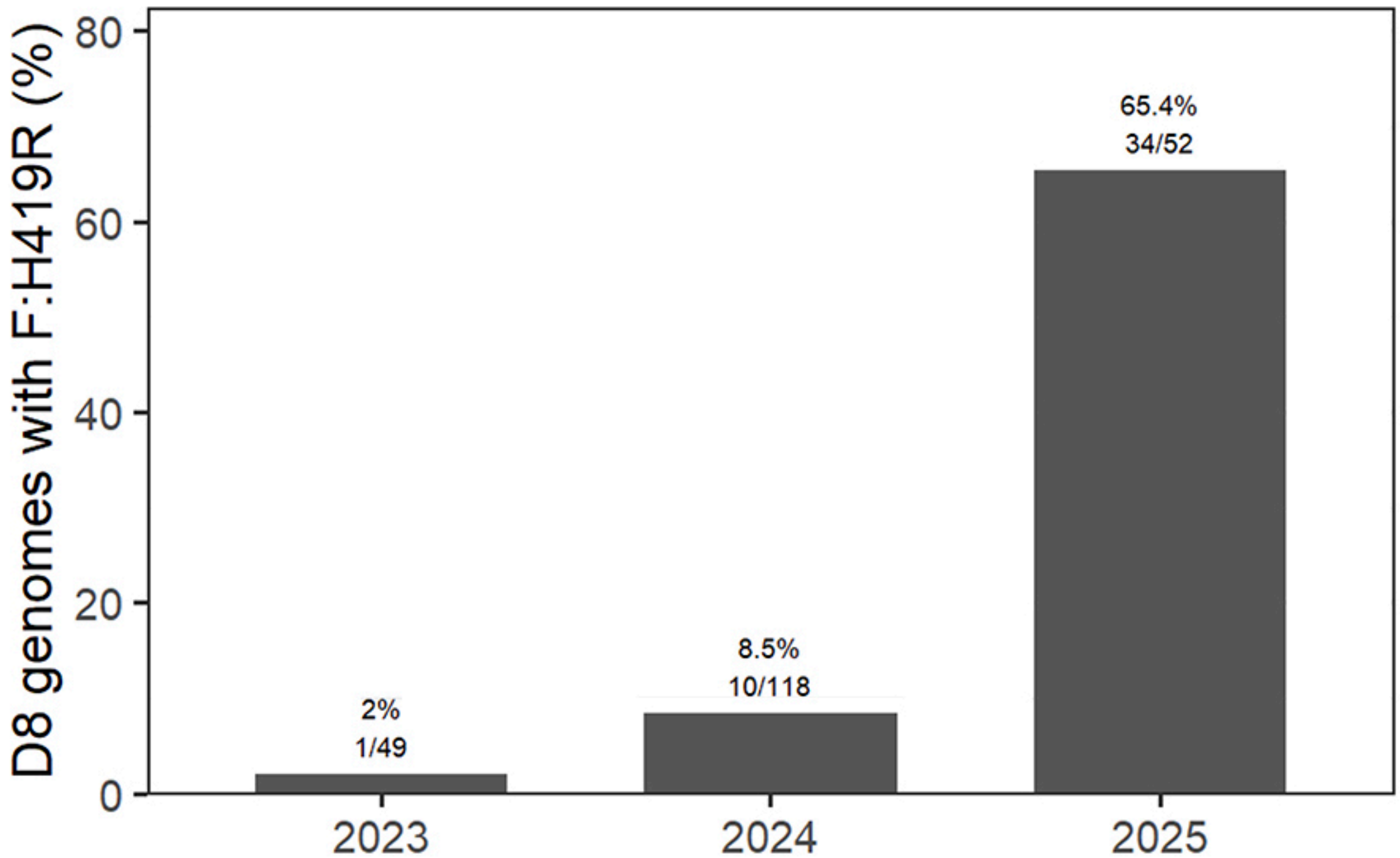

Appendix Figure 3. Detection of F:H419R among publicly available genotype D8 whole-genome sequences with sufficient Fusion gene coverage, by year, 2023–2025. Percentages are shown above bars with the number of F:H419R-positive genomes and total D8 genomes analyzed. NCBI data were accessed on 2025-09-24.

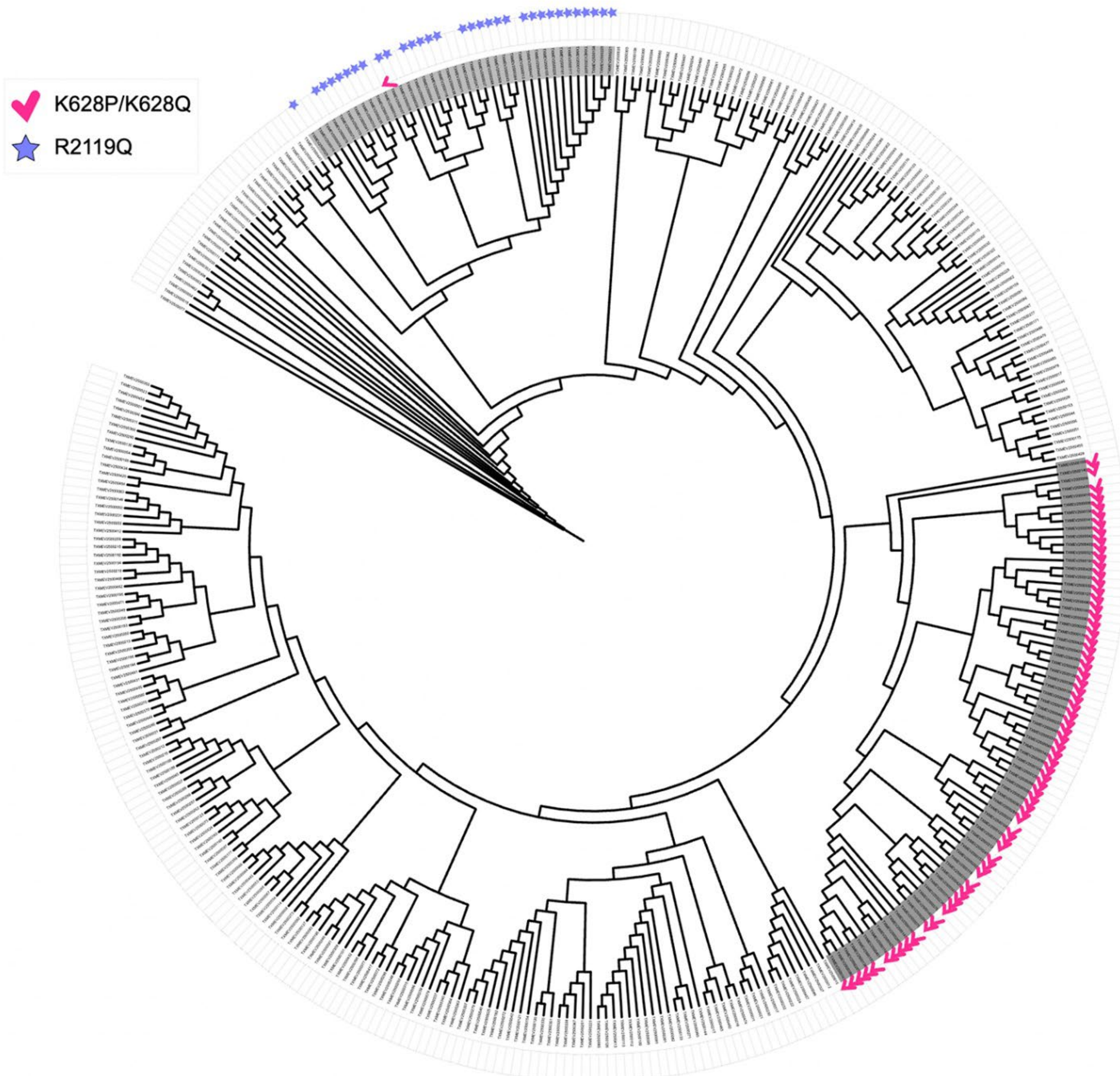

Appendix Figure 4. Polymerase-gene codons identified by selection analysis and used as subclade markers. Selection analysis identified two polymerase-gene codons with evidence of selection. These codons were treated only as subclade markers and were not interpreted as evidence of immune escape, functional adaptation, altered transmission, or clinical outcome.

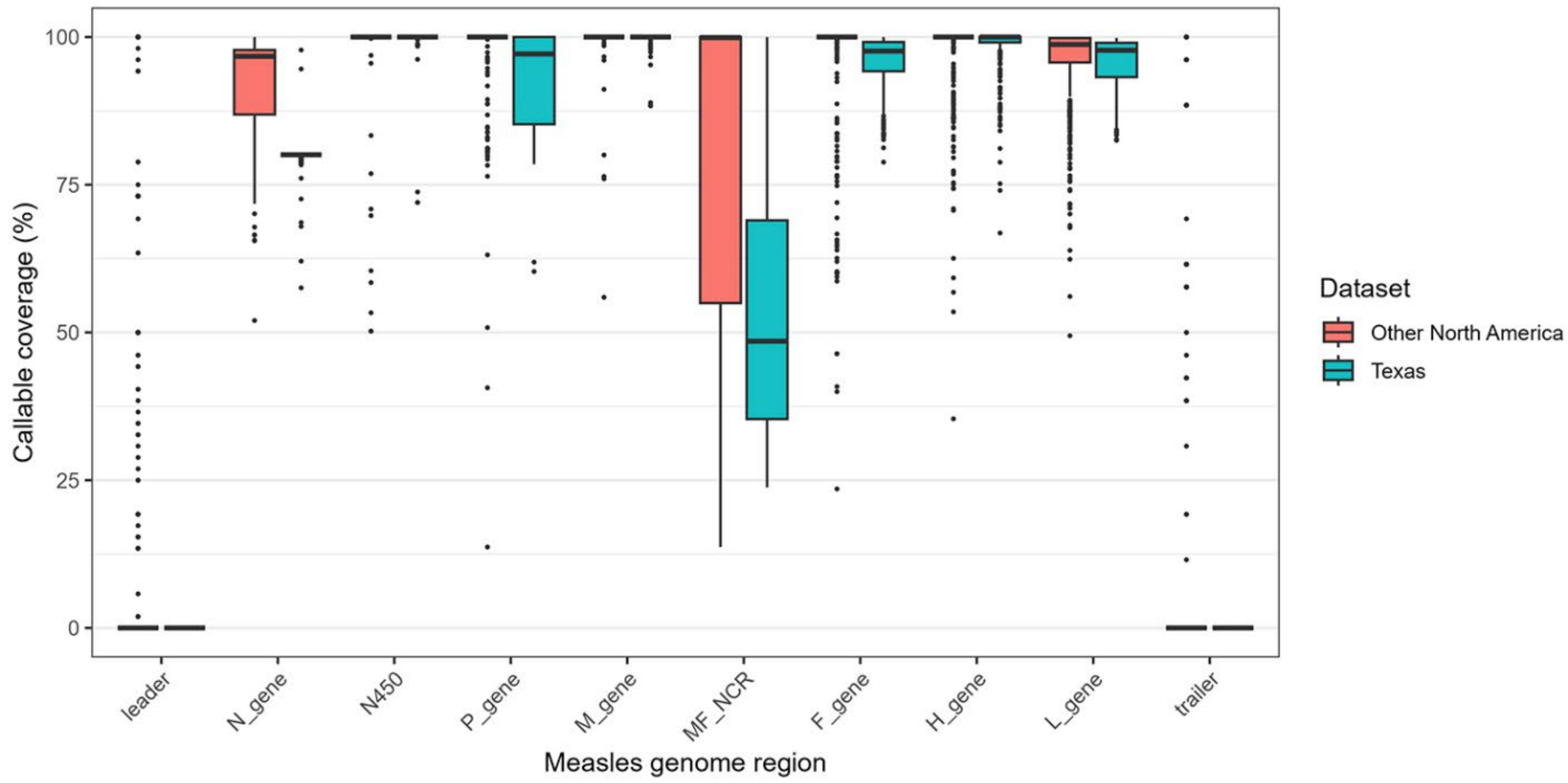

Appendix Figure 5. Callable coverage by measles virus genome region for Texas and other North American contextual genomes. Callable coverage percentages across measles virus genomic regions, calculated by mapping KJ018971.2 reference coordinates to alignment positions and measuring the proportion of reference-homologous sites with A/C/G/T calls. Texas genomes are compared with other North American genomes included in the contextual phylogenetic analysis.

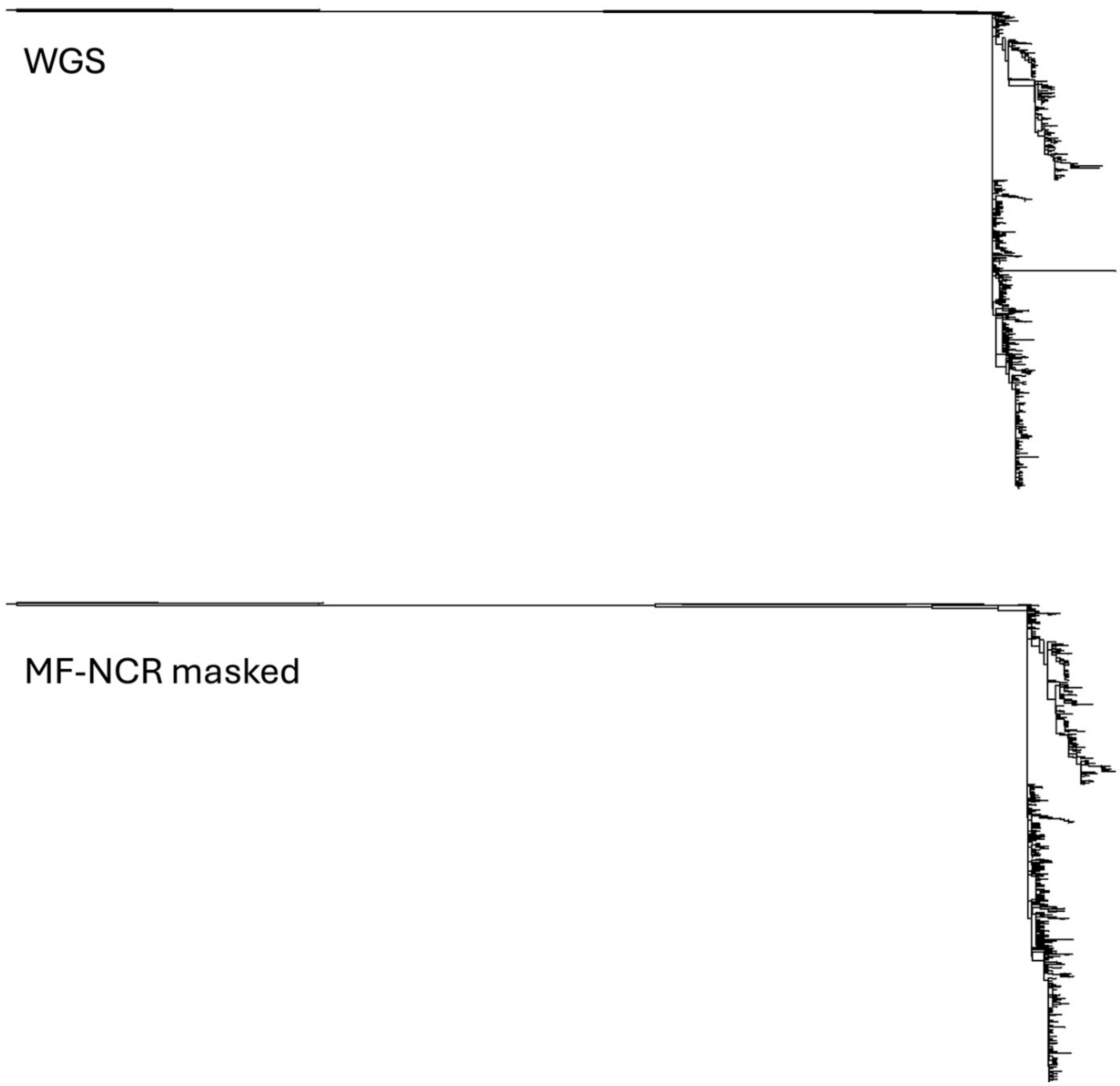

Appendix Figure 6. Sensitivity phylogeny of the North American measles genomes after masking MF-NCR. Maximum-likelihood phylogenies reconstructed from the whole-genome alignment and from an alignment with MF-NCR positions masked. The major clustering pattern was retained after MF-NCR masking, indicating that uneven MF-NCR coverage did not account for the broad phylogenetic structure.

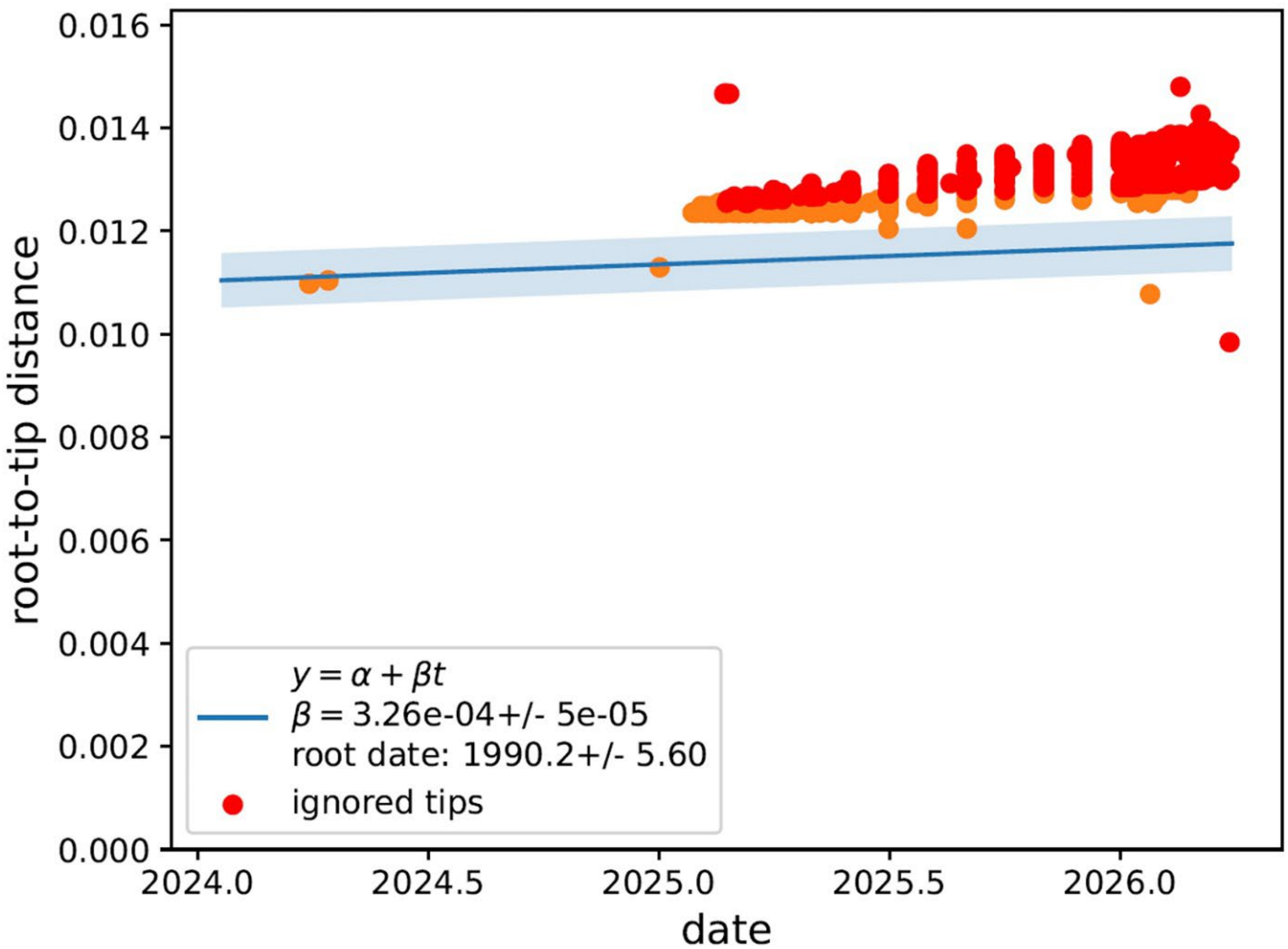

Appendix Figure 7. TreeTime molecular-clock fit for the North American contextual genotype D8 dataset. Root-to-tip genetic distance was plotted against sampling date for the North American contextual genotype D8 dataset (n = 1,771) during TreeTime time-scaled phylogenetic inference. Tips identified by TreeTime as molecular-clock outliers were excluded from clock-rate fitting. The fitted TreeTime clock rate was 3.26×10^{-4} substitutions/site/year, with $R^2 = 0.60$. These TreeTime diagnostics were used to evaluate temporal fit under the TreeTime model and are distinct from the Clockor2 strict-clock root-to-tip regression diagnostics summarized in Appendix Table 4.
